# Biological, Psychological, and Social Determinants of Depression: A Review of Recent Literature

**DOI:** 10.3390/brainsci11121633

**Published:** 2021-12-10

**Authors:** Olivia Remes, João Francisco Mendes, Peter Templeton

**Affiliations:** 1 Institute for Manufacturing, University of Cambridge, Cambridge CB3 0FS, UK; 2 NOVA Medical School, Universidade NOVA de Lisboa, 1099-085 Lisbon, Portugal; jfc49@cam.ac.uk; 3 IfM Engage Limited, Institute for Manufacturing, University of Cambridge, Cambridge CB3 0FS, UK; pwt23@cam.ac.uk; 4 The William Templeton Foundation for Young People’s Mental Health (YPMH), Cambridge CB2 0AH, UK

**Keywords:** depression, major depressive disorder, risk factor, protective factor, determinant, review

## Abstract

Depression is one of the leading causes of disability, and, if left unmanaged, it can increase the risk for suicide. The evidence base on the determinants of depression is fragmented, which makes the interpretation of the results across studies difficult. The objective of this study is to conduct a thorough synthesis of the literature assessing the biological, psychological, and social determinants of depression in order to piece together the puzzle of the key factors that are related to this condition. Titles and abstracts published between 2017 and 2020 were identified in PubMed, as well as Medline, Scopus, and PsycInfo. Key words relating to biological, social, and psychological determinants as well as depression were applied to the databases, and the screening and data charting of the documents took place. We included 470 documents in this literature review. The findings showed that there are a plethora of risk and protective factors (relating to biological, psychological, and social determinants) that are related to depression; these determinants are interlinked and influence depression outcomes through a web of causation. In this paper, we describe and present the vast, fragmented, and complex literature related to this topic. This review may be used to guide practice, public health efforts, policy, and research related to mental health and, specifically, depression.

## 1. Introduction

Depression is one of the most common mental health issues, with an estimated prevalence of 5% among adults [1,2]. Symptoms may include anhedonia, feelings of worthlessness, concentration and sleep difficulties, and suicidal ideation. According to the World Health Organization, depression is a leading cause of disability; research shows that it is a burdensome condition with a negative impact on educational trajectories, work performance, and other areas of life [1,3]. Depression can start early in the lifecourse and, if it remains unmanaged, may increase the risk for substance abuse, chronic conditions, such as cardiovascular disease, and premature mortality [4,5,6,7,8].

Treatment for depression exists, such as pharmacotherapy, cognitive behavioural therapy, and other modalities. A meta-analysis of randomized, placebo-controlled trials of patients shows that 56–60% of people respond well to active treatment with antidepressants (selective serotonin reuptake inhibitors, tricyclic antidepressants) [9]. However, pharmacotherapy may be associated with problems, such as side-effects, relapse issues, a potential duration of weeks until the medication starts working, and possible limited efficacy in mild cases [10,11,12,13,14]. Psychotherapy is also available, but access barriers can make it difficult for a number of people to get the necessary help. 

Studies on depression have increased significantly over the past few decades. However, the literature remains fragmented and the interpretation of heterogeneous findings across studies and between fields is difficult. The cross-pollination of ideas between disciplines, such as genetics, neurology, immunology, and psychology, is limited. Reviews on the determinants of depression have been conducted, but they either focus exclusively on a particular set of determinants (ex. genetic risk factors [15]) or population sub-group (ex. children and adolescents [16]) or focus on characteristics measured predominantly at the individual level (ex. focus on social support, history of depression [17]) without taking the wider context (ex. area-level variables) into account. An integrated approach paying attention to key determinants from the biological, psychological, and social spheres, as well as key themes, such as the lifecourse perspective, enables clinicians and public health authorities to develop tailored, person-centred approaches.

The primary aim of this literature review: to address the aforementioned challenges, we have synthesized recent research on the biological, psychological, and social determinants of depression and we have reviewed research from fields including genetics, immunology, neurology, psychology, public health, and epidemiology, among others.

The subsidiary aim: we have paid special attention to important themes, including the lifecourse perspective and interactions between determinants, to guide further efforts by public health and medical professionals.

This literature review can be used as an evidence base by those in public health and the clinical setting and can be used to inform targeted interventions.

## 2. Materials and Methods

We conducted a review of the literature on the biological, psychological, and social determinants of depression in the last 4 years. We decided to focus on these determinants after discussions with academics (from the Manchester Metropolitan University, University of Cardiff, University of Colorado, Boulder, University of Cork, University of Leuven, University of Texas), charity representatives, and people with lived experience at workshops held by the University of Cambridge in 2020. In several aspects, we attempted to conduct this review according to PRISMA guidelines [18].

The inclusion and exclusion criteria are the following:

- Inclusion criteria
  - We included documents, such as primary studies, literature reviews, systematic reviews, meta-analyses, reports, and commentaries on the determinants of depression. The determinants refer to variables that appear to be linked to the development of depression, such as physiological factors (e.g., the nervous system, genetics), but also factors that are further away or more distal to the condition. Determinants may be risk or protective factors, and individual- or wider-area-level variables.
  - We focused on major depressive disorder, treatment-resistant depression, dysthymia, depressive symptoms, poststroke depression, perinatal depression, as well as depressive-like behaviour (common in animal studies), among others.
  - We included papers regardless of the measurement methods of depression.
  - We included papers that focused on human and/or rodent research.
  - This review focused on articles written in the English language.
  - Documents published between 2017–2020 were captured to provide an understanding of the latest research on this topic.
- Exclusion criteria
  - Studies that assessed depression as a comorbidity or secondary to another disorder.
  - Studies that did not focus on rodent and/or human research.
  - Studies that focused on the treatment of depression. We made this decision, because this is an in-depth topic that would warrant a separate stand-alone review.

1. Next, we searched PubMed (2017–2020) using keywords related to depression and determinants. Appendix A contains the search strategy used. We also conducted focused searches in Medline, Scopus, and PsycInfo (2017–2020).
2. Once the documents were identified through the databases, the inclusion and exclusion criteria were applied to the titles and abstracts. Screening of documents was conducted by O.R., and a subsample was screened by J.M.; any discrepancies were resolved through a communication process.
3. The full texts of documents were retrieved, and the inclusion and exclusion criteria were again applied. A subsample of documents underwent double screening by two authors (O.R., J.M.); again, any discrepancies were resolved through communication.
4. The final list of references that were eligible and met the inclusion criteria was included in a data charting form.
  a. A data charting form was created to capture the data elements of interest, including the authors, titles, determinants (biological, psychological, social), and the type of depression assessed by the research (e.g., major depression, depressive symptoms, depressive behaviour).
  b. The data charting form was piloted on a subset of documents, and refinements to it were made. The data charting form was created with the data elements described above and tested in 20 studies to determine whether refinements in the wording or language were needed.
  c. Data charting was conducted on the documents.
  d. Narrative analysis was conducted on the data charting table to identify key themes. When a particular finding was noted more than once, it was logged as a potential theme, with a review of these notes yielding key themes that appeared on multiple occasions. When key themes were identified, one researcher (O.R.) reviewed each document pertaining to that theme and derived concepts (key determinants and related outcomes). This process (a subsample) was verified by a second author (J.M.), and the two authors resolved any discrepancies through communication. Key themes were also checked as to whether they were of major significance to public mental health and at the forefront of public health discourse according to consultations we held with stakeholders from the Manchester Metropolitan University, University of Cardiff, University of Colorado, Boulder, University of Cork, University of Leuven, University of Texas, charity representatives, and people with lived experience at workshops held by the University of Cambridge in 2020.

We condensed the extensive information gleaned through our review into short summaries (with key points boxes for ease of understanding and interpretation of the data).

## 3. Results

Through the searches, 6335 documents, such as primary studies, literature reviews, systematic reviews, meta-analyses, reports, and commentaries, were identified. After applying the inclusion and exclusion criteria, 470 papers were included in this review (Supplementary Table S1). We focused on aspects related to biological, psychological, and social determinants of depression (examples of determinants and related outcomes are provided under each of the following sections.

### 3.1. Biological Factors

The following aspects will be discussed in this section: physical health conditions; then specific biological factors, including genetics; the microbiome; inflammatory factors; stress and hypothalamic–pituitary–adrenal (HPA) axis dysfunction, and the kynurenine pathway. Finally, aspects related to cognition will also be discussed in the context of depression.

#### 3.1.1. Physical Health Conditions

Studies on physical health conditions—key points:

- The presence of a physical health condition can increase the risk for depression
- Psychological evaluation in physically sick populations is needed
- There is large heterogeneity in study design and measurement; this makes the comparison of findings between and across studies difficult

A number of studies examined the links between the outcome of depression and physical health-related factors, such as bladder outlet obstruction, cerebral atrophy, cataract, stroke, epilepsy, body mass index and obesity, diabetes, urinary tract infection, forms of cancer, inflammatory bowel disorder, glaucoma, acne, urea accumulation, cerebral small vessel disease, traumatic brain injury, and disability in multiple sclerosis [19,20,21,22,23,24,25,26,27,28,29,30,31,32,33,34,35,36,37,38,39,40,41,42,43,44,45,46,47,48,49,50,51,52,53,54,55,56,57,58,59,60,61,62,63,64,65,66,67,68,69,70,71]. For example, bladder outlet obstruction has been linked to inflammation and depressive behaviour in rodent research [24]. The presence of head and neck cancer also seemed to be related to an increased risk for depressive disorder [45]. Gestational diabetes mellitus has been linked to depressive symptoms in the postpartum period (but no association has been found with depression in the third pregnancy trimester) [50], and a plethora of other such examples of relationships between depression and physical conditions exist. As such, the assessment of psychopathology and the provision of support are necessary in individuals of ill health [45]. Despite the large evidence base on physical health-related factors, differences in study methodology and design, the lack of standardization when it comes to the measurement of various physical health conditions and depression, and heterogeneity in the study populations makes it difficult to compare studies [50].

The next subsections discuss specific biological factors, including genetics; the microbiome; inflammatory factors; stress and hypothalamic–pituitary–adrenal (HPA) axis dysfunction, and the kynurenine pathway; and aspects related to cognition.

#### 3.1.2. Genetics

Studies on genetics—key points:

There were associations between genetic factors and depression; for example:

- The brain-derived neurotrophic factor (BDNF) plays an important role in depression
- Links exist between major histocompatibility complex region genes, as well as various gene polymorphisms and depression
- Single nucleotide polymorphisms (SNPs) of genes involved in the tryptophan catabolites pathway are of interest in relation to depression

A number of genetic-related factors, genomic regions, polymorphisms, and other related aspects have been examined with respect to depression [61,72,73,74,75,76,77,78,79,80,81,82,83,84,85,86,87,88,89,90,91,92,93,94,95,96,97,98,99,100,101,102,103,104,105,106,107,108,109,110,111,112,113,114,115,116,117,118,119,120,121,122,123,124,125,126,127,128,129,130,131,132,133,134,135,136,137,138,139,140]. The influence of BDNF in relation to depression has been amply studied [117,118,141,142,143]. Research has shown associations between depression and BDNF (as well as candidate SNPs of the BDNF gene, polymorphisms of the BDNF gene, and the interaction of these polymorphisms with other determinants, such as stress) [129,144,145]. Specific findings have been reported: for example, a study reported a link between the BDNF rs6265 allele (A) and major depressive disorder [117].

Other research focused on major histocompatibility complex region genes, endocannabinoid receptor gene polymorphisms, as well as tissue-specific genes and gene co-expression networks and their links to depression [99,110,112]. The SNPs of genes involved in the tryptophan catabolites pathway have also been of interest when studying the pathogenesis of depression.

The results from genetics studies are compelling; however, the findings remain mixed. One study indicated no support for depression candidate gene findings [122]. Another study found no association between specific polymorphisms and major depressive disorder [132]. As such, further research using larger samples is needed to corroborate the statistically significant associations reported in the literature.

#### 3.1.3. Microbiome

Studies on the microbiome—key points:

- The gut bacteria and the brain communicate via both direct and indirect pathways called the gut-microbiota-brain axis (the bidirectional communication networks between the central nervous system and the gastrointestinal tract; this axis plays an important role in maintaining homeostasis).
- A disordered microbiome can lead to inflammation, which can then lead to depression
- There are possible links between the gut microbiome, host liver metabolism, brain inflammation, and depression

The common themes of this review have focused on the microbiome/microbiota or gut metabolome [146,147,148,149,150,151,152,153,154,155,156,157,158,159,160,161], the microbiota-gut-brain axis, and related factors [152,162,163,164,165,166,167]. When there is an imbalance in the intestinal bacteria, this can interfere with emotional regulation and contribute to harmful inflammatory processes and mood disorders [148,151,153,155,157]. Rodent research has shown that there may be a bidirectional association between the gut microbiota and depression: a disordered gut microbiota can play a role in the onset of this mental health problem, but, at the same time, the existence of stress and depression may also lead to a lower level of richness and diversity in the microbiome [158].

Research has also attempted to disentangle the links between the gut microbiome, host liver metabolism, brain inflammation, and depression, as well as the role of the ratio of lactobacillus to clostridium [152]. The literature has also examined the links between medication, such as antibiotics, and mood and behaviour, with the findings showing that antibiotics may be related to depression [159,168]. The links between the microbiome and depression are complex, and further studies are needed to determine the underpinning causal mechanisms.

#### 3.1.4. Inflammation

Studies on inflammation—key points:

- Pro-inflammatory cytokines are linked to depression
- Pro-inflammatory cytokines, such as the tumour necrosis factor (TNF)-alpha, may play an important role
- Different methods of measurement are used, making the comparison of findings across studies difficult

Inflammation has been a theme in this literature review [60,161,164,169,170,171,172,173,174,175,176,177,178,179,180,181,182,183,184]. The findings show that raised levels of inflammation (because of factors such as pro-inflammatory cytokines) have been associated with depression [60,161,174,175,178]. For example, pro-inflammatory cytokines, such as tumour necrosis factor (TNF)-alpha, have been linked to depression [185]. Various determinants, such as early life stress, have also been linked to systemic inflammation, and this can increase the risk for depression [186].

Nevertheless, not everyone with elevated inflammation develops depression; therefore, this is just one route out of many linked to pathogenesis. Despite the compelling evidence reported with respect to inflammation, it is difficult to compare the findings across studies because of different methods used to assess depression and its risk factors.

#### 3.1.5. Stress and HPA Axis Dysfunction

Studies on stress and HPA axis dysfunction—key points:

- Stress is linked to the release of proinflammatory factors
- The dysregulation of the HPA axis is linked to depression
- Determinants are interlinked in a complex web of causation

Stress was studied in various forms in rodent populations and humans [144,145,155,174,176,180,185,186,187,188,189,190,191,192,193,194,195,196,197,198,199,200,201,202,203,204,205,206,207,208,209,210,211].

Although this section has some overlap with others (as is to be expected because all of these determinants and body systems are interlinked), a number of studies have focused on the impact of stress on mental health. Stress has been mentioned in the literature as a risk factor of poor mental health and has emerged as an important determinant of depression. The effects of this variable are wide-ranging, and a short discussion is warranted.

Stress has been linked to the release of inflammatory factors, as well as the development of depression [204]. When the stress is high or lasts for a long period of time, this may negatively impact the brain. Chronic stress can impact the dendrites and synapses of various neurons, and may be implicated in the pathway leading to major depressive disorder [114]. As a review by Uchida et al. indicates, stress may be associated with the “dysregulation of neuronal and synaptic plasticity” [114]. Even in rodent studies, stress has a negative impact: chronic and unpredictable stress (and other forms of tension or stress) have been linked to unusual behaviour and depression symptoms [114].

The depression process and related brain changes, however, have also been linked to the hyperactivity or dysregulation of the HPA axis [127,130,131,182,212]. One review indicates that a potential underpinning mechanism of depression relates to “HPA axis abnormalities involved in chronic stress” [213]. There is a complex relationship between the HPA axis, glucocorticoid receptors, epigenetic mechanisms, and psychiatric sequelae [130,212].

In terms of the relationship between the HPA axis and stress and their influence on depression, the diathesis–stress model offers an explanation: it could be that early stress plays a role in the hyperactivation of the HPA axis, thus creating a predisposition “towards a maladaptive reaction to stress”. When this predisposition then meets an acute stressor, depression may ensue; thus, in line with the diathesis–stress model, a pre-existing vulnerability and stressor can create fertile ground for a mood disorder [213]. An integrated review by Dean and Keshavan [213] suggests that HPA axis hyperactivity is, in turn, related to other determinants, such as early deprivation and insecure early attachment; this again shows the complex web of causation between the different determinants.

#### 3.1.6. Kynurenine Pathway

Studies on the kynurenine pathway—key points:

- The kynurenine pathway is linked to depression
- Indolamine 2,3-dioxegenase (IDO) polymorphisms are linked to postpartum depression

The kynurenine pathway was another theme that emerged in this review [120,178,181,184,214,215,216,217,218,219,220,221]. The kynurenine pathway has been implicated not only in general depressed mood (inflammation-induced depression) [184,214,219] but also postpartum depression [120]. When the kynurenine metabolism pathway is activated, this results in metabolites, which are neurotoxic.

A review by Jeon et al. notes a link between the impairment of the kynurenine pathway and inflammation-induced depression (triggered by treatment for various physical diseases, such as malignancy). The authors note that this could represent an important opportunity for immunopharmacology [214]. Another review by Danzer et al. suggests links between the inflammation-induced activation of indolamine 2,3-dioxegenase (the enzyme that converts tryptophan to kynurenine), the kynurenine metabolism pathway, and depression, and also remarks about the “opportunities for treatment of inflammation-induced depression” [184].

#### 3.1.7. Cognition

Studies on cognition and the brain—key points:

- Cognitive decline and cognitive deficits are linked to increased depression risk
- Cognitive reserve is important in the disability/depression relationship
- Family history of cognitive impairment is linked to depression

A number of studies have focused on the theme of cognition and the brain. The results show that factors, such as low cognitive ability/function, cognitive vulnerability, cognitive impairment or deficits, subjective cognitive decline, regression of dendritic branching and hippocampal atrophy/death of hippocampal cells, impaired neuroplasticity, and neurogenesis-related aspects, have been linked to depression [131,212,222,223,224,225,226,227,228,229,230,231,232,233,234,235,236,237,238,239]. The cognitive reserve appears to act as a moderator and can magnify the impact of certain determinants on poor mental health. For example, in a study in which participants with multiple sclerosis also had low cognitive reserve, disability was shown to increase the risk for depression [63]. Cognitive deficits can be both causal and resultant in depression. A study on individuals attending outpatient stroke clinics showed that lower scores in cognition were related to depression; thus, cognitive impairment appears to be associated with depressive symptomatology [226]. Further, Halahakoon et al. [222] note a meta-analysis [240] that shows that a family history of cognitive impairment (in first degree relatives) is also linked to depression.

In addition to cognitive deficits, low-level cognitive ability [231] and cognitive vulnerability [232] have also been linked to depression. While cognitive impairment may be implicated in the pathogenesis of depressive symptoms [222], negative information processing biases are also important; according to the ‘cognitive neuropsychological’ model of depression, negative affective biases play a central part in the development of depression [222,241]. Nevertheless, the evidence on this topic is mixed and further work is needed to determine the underpinning mechanisms between these states.

### 3.2. Psychological Factors

Studies on psychological factors—key points:

- There are many affective risk factors linked to depression
- Determinants of depression include negative self-concept, sensitivity to rejection, neuroticism, rumination, negative emotionality, and others

A number of studies have been undertaken on the psychological factors linked to depression (including mastery, self-esteem, optimism, negative self-image, current or past mental health conditions, and various other aspects, including neuroticism, brooding, conflict, negative thinking, insight, cognitive fusion, emotional clarity, rumination, dysfunctional attitudes, interpretation bias, and attachment style) [66,128,140,205,210,228,235,242,243,244,245,246,247,248,249,250,251,252,253,254,255,256,257,258,259,260,261,262,263,264,265,266,267,268,269,270,271,272,273,274,275,276,277,278,279,280,281,282,283,284,285,286,287,288,289,290]. Determinants related to this condition include low self-esteem and shame, among other factors [269,270,275,278]. Several emotional states and traits, such as neuroticism [235,260,271,278], negative self-concept (with self-perceptions of worthlessness and uselessness), and negative interpretation or attention biases have been linked to depression [261,271,282,283,286]. Moreover, low emotional clarity has been associated with depression [267]. When it comes to the severity of the disorder, it appears that meta-emotions (“emotions that occur in response to other emotions (e.g., guilt about anger)” [268]) have a role to play in depression [268].

A determinant that has received much attention in mental health research concerns rumination. Rumination has been presented as a mediator but also as a risk factor for depression [57,210,259]. When studied as a risk factor, it appears that the relationship of rumination with depression is mediated by variables that include limited problem-solving ability and insufficient social support [259]. However, rumination also appears to act as a mediator: for example, this variable (particularly brooding rumination) lies on the causal pathway between poor attention control and depression [265]. This shows that determinants may present in several forms: as moderators or mediators, risk factors or outcomes, and this is why disentangling the relationships between the various factors linked to depression is a complex task.

The psychological determinants are commonly researched variables in the mental health literature. A wide range of factors have been linked to depression, such as the aforementioned determinants, but also: (low) optimism levels, maladaptive coping (such as avoidance), body image issues, and maladaptive perfectionism, among others [269,270,272,273,275,276,279,285,286]. Various mechanisms have been proposed to explain the way these determinants increase the risk for depression. One of the underpinning mechanisms linking the determinants and depression concerns coping. For example, positive fantasy engagement, cognitive biases, or personality dispositions may lead to emotion-focused coping, such as brooding, and subsequently increase the risk for depression [272,284,287]. Knowing the causal mechanisms linking the determinants to outcomes provides insight for the development of targeted interventions.

### 3.3. Social Determinants

Studies on social determinants—key points:

- Social determinants are the conditions in the environments where people are born, live, learn, work, play, etc.; these influence (mental) health [291]
- There are many social determinants linked to depression, such as sociodemographics, social support, adverse childhood experiences
- Determinants can be at the individual, social network, community, and societal levels

Studies also focused on the social determinants of (mental) health; these are the conditions in which people are born, live, learn, work, play, and age, and have a significant influence on wellbeing [291]. Factors such as age, social or socioeconomic status, social support, financial strain and deprivation, food insecurity, education, employment status, living arrangements, marital status, race, childhood conflict and bullying, violent crime exposure, abuse, discrimination, (self)-stigma, ethnicity and migrant status, working conditions, adverse or significant life events, illiteracy or health literacy, environmental events, job strain, and the built environment have been linked to depression, among others [52,133,235,236,239,252,269,280,292,293,294,295,296,297,298,299,300,301,302,303,304,305,306,307,308,309,310,311,312,313,314,315,316,317,318,319,320,321,322,323,324,325,326,327,328,329,330,331,332,333,334,335,336,337,338,339,340,341,342,343,344,345,346,347,348,349,350,351,352,353,354,355,356,357,358,359,360,361,362,363,364,365,366,367,368,369,370,371]. Social support and cohesion, as well as structural social capital, have also been identified as determinants [140,228,239,269,293,372,373,374,375,376,377,378,379]. In a study, part of the findings showed that low levels of education have been shown to be linked to post-stroke depression (but not severe or clinical depression outcomes) [299]. A study within a systematic review indicated that having only primary education was associated with a higher risk of depression compared to having secondary or higher education (although another study contrasted this finding) [296]. Various studies on socioeconomic status-related factors have been undertaken [239,297]; the research has shown that a low level of education is linked to depression [297]. Low income is also related to depressive disorders [312]. By contrast, high levels of education and income are protective [335].

A group of determinants touched upon by several studies included adverse childhood or early life experiences: ex. conflict with parents, early exposure to traumatic life events, bullying and childhood trauma were found to increase the risk of depression (ex. through pathways, such as inflammation, interaction effects, or cognitive biases) [161,182,258,358,362,380].

Gender-related factors were also found to play an important role with respect to mental health [235,381,382,383,384,385]. Gender inequalities can start early on in the lifecourse, and women were found to be twice as likely to have depression as men. Gender-related factors were linked to cognitive biases, resilience and vulnerabilities [362,384].

Determinants can impact mental health outcomes through underpinning mechanisms. For example, harmful determinants can influence the uptake of risk behaviours. Risk behaviours, such as sedentary behaviour, substance abuse and smoking/nicotine exposure, have been linked to depression [226,335,355,385,386,387,388,389,390,391,392,393,394,395,396,397,398,399,400,401]. Harmful determinants can also have an impact on diet. Indeed, dietary aspects and diet components (ex. vitamin D, folate, selenium intake, iron, vitamin B12, vitamin K, fiber intake, zinc) as well as diet-related inflammatory potential have been linked to depression outcomes [161,208,236,312,396,402,403,404,405,406,407,408,409,410,411,412,413,414,415,416,417,418,419,420,421,422,423,424,425,426,427,428]. A poor diet has been linked to depression through mechanisms such as inflammation [428].

Again, it is difficult to constrict diet to the ‘social determinants of health’ category as it also relates to inflammation (biological determinants) and could even stand alone as its own category. Nevertheless, all of these factors are interlinked and influence one another in a complex web of causation, as mentioned elsewhere in the paper.

Supplementary Figure S1 contains a representation of key determinants acting at various levels: the individual, social network, community, and societal levels. The determinants have an influence on risk behaviours, and this, in turn, can affect the mood (i.e., depression), body processes (ex. can increase inflammation), and may negatively influence brain structure and function.

### 3.4. Others

Studies on ‘other’ determinants—key points:

- A number of factors are related to depression
- These may not be as easily categorized as the other determinants in this paper

A number of factors arose in this review that were related to depression; it was difficult to place these under a specific heading above, so this ‘other’ category was created. A number of these could be sorted under the ‘social determinants of depression’ category. For example, being exposed to deprivation, hardship, or adversity may increase the risk for air pollution exposure and nighttime shift work, among others, and the latter determinants have been found to increase the risk for depression. Air pollution could also be regarded as an ecologic-level (environmental) determinant of mental health.

Nevertheless, we have decided to leave these factors in a separate category (because their categorization may not be as immediately clear-cut as others), and these factors include: low-level light [429], weight cycling [430], water contaminants [431], trade [432], air pollution [433,434], program-level variables (ex. feedback and learning experience) [435], TV viewing [436], falls [437], various other biological factors [116,136,141,151,164,182,363,364,438,439,440,441,442,443,444,445,446,447,448,449,450,451,452,453,454,455,456,457,458,459,460,461,462,463,464,465,466,467,468,469], mobile phone use [470], ultrasound chronic exposure [471], nighttime shift work [472], work accidents [473], therapy enrollment [226], and exposure to light at night [474].

## 4. Cross-Cutting Themes

### 4.1. Lifecourse Perspective

Studies on the lifecourse perspective—key points:

- Early life has an importance on mental health
- Stress has been linked to depression
- In old age, the decline in social capital is important

Trajectories and life events are important when it comes to the lifecourse perspective. Research has touched on the influence of prenatal or early life stress on an individual’s mental health trajectory [164,199,475]. Severe stress that occurs in the form of early-life trauma has also been associated with depressive symptoms [362,380]. It may be that some individuals exposed to trauma develop thoughts of personal failure, which then serve as a catalyst of depression [380].

At the other end of the life trajectory—old age—specific determinants have been linked to an increased risk for depression. Older people are at a heightened risk of losing their social networks, and structural social capital has been identified as important in relation to depression in old age [293].

### 4.2. Gene–Environment Interactions

Studies on gene–environment interactions—key points:

- The environment and genetics interact to increase the risk of depression
- The etiology of depression is multifactorial
- Adolescence is a time of vulnerability

A number of studies have touched on gene–environment interactions [72,77,82,119,381,476,477,478,479,480,481]. The interactions between genetic factors and determinants, such as negative life events (ex. relationship and social difficulties, serious illness, unemployment and financial crises) and stressors (ex. death of spouse, minor violations of law, neighbourhood socioeconomic status) have been studied in relation to depression [82,135,298,449,481]. A study reported an interaction of significant life events with functional variation in the serotonin-transporter-linked polymorphic region (5-HTTLPR) allele type (in the context of multiple sclerosis) and linked this to depression [361], while another reported an interaction between stress and 5-HTTLPR in relation to depression [480]. Other research reported that the genetic variation of HPA-axis genes has moderating effects on the relationship between stressors and depression [198]. Another study showed that early-life stress interacts with gene variants to increase the risk for depression [77].

Adolescence is a time of vulnerability [111,480]. Perceived parental support has been found to interact with genes (GABRR1, GABRR2), and this appears to be associated with depressive symptoms in adolescence [480]. It is important to pay special attention to critical periods in the lifecourse so that adequate support is provided to those who are most vulnerable.

The etiology of depression is multifactorial, and it is worthwhile to examine the interaction between multiple factors, such as epigenetic, genetic, and environmental factors, in order to truly understand this mental health condition. Finally, taking into account critical periods of life when assessing gene–environment interactions is important for developing targeted interventions.

## 5. Discussion

Depression is one of the most common mental health conditions, and, if left untreated, it can increase the risk for substance abuse, anxiety disorders, and suicide. In the past 20 years, a large number of studies on the risk and protective factors of depression have been undertaken in various fields, such as genetics, neurology, immunology, and epidemiology. However, there are limitations associated with the extant evidence base. The previous syntheses on depression are limited in scope and focus exclusively on social or biological factors, population sub-groups, or examine depression as a comorbidity (rather than an independent disorder). The research on the determinants and causal pathways of depression is fragmentated and heterogeneous, and this has not helped to stimulate progress when it comes to the prevention and intervention of this condition—specifically unravelling the complexity of the determinants related to this condition and thus refining the prevention and intervention methods.

The scope of this paper was to bring together the heterogeneous, vast, and fragmented literature on depression and paint a picture of the key factors that contribute to this condition. The findings from this review show that there are important themes when it comes to the determinants of depression, such as: the microbiome, dysregulation of the HPA axis, inflammatory reactions, the kynurenine pathway, as well as psychological and social factors. It may be that physical factors are proximal determinants of depression, which, in turn, are acted on by more distal social factors, such as deprivation, environmental events, and social capital.

The Marmot Report [291], the World Health Organization [482], and Compton et al. [483] highlight that the most disadvantaged segments of society are suffering (the socioeconomic context is important), and this inequality in resources has translated to inequality in mental health outcomes [483]. To tackle the issue of egalitarianism and restore equality in the health between the groups, the social determinants need to be addressed [483]. A wide range of determinants of mental health have been identified in the literature: age, gender, ethnicity, family upbringing and early attachment patterns, social support, access to food, water and proper nutrition, and community factors. People spiral downwards because of individual- and societal-level circumstances; therefore, these circumstances along with the interactions between the determinants need to be considered.

Another important theme in the mental health literature is the lifecourse perspective. This shows that the timing of events has significance when it comes to mental health. Early life is a critical period during the lifespan at which cognitive processes develop. Exposure to harmful determinants, such as stress, during this period can place an individual on a trajectory of depression in adulthood or later life. When an individual is exposed to harmful determinants during critical periods and is also genetically predisposed to depression, the risk for the disorder can be compounded. This is why aspects such as the lifecourse perspective and gene–environment interactions need to be taken into account. Insight into this can also help to refine targeted interventions.

A number of interventions for depression have been developed or recommended, addressing, for example, the physical factors described here and lifestyle modifications. Interventions targeting various factors, such as education and socioeconomic status, are needed to help prevent and reduce the burden of depression. Further research on the efficacy of various interventions is needed. Additional studies are also needed on each of the themes described in this paper, for example: the biological factors related to postpartum depression [134], and further work is needed on depression outcomes, such as chronic, recurrent depression [452]. Previous literature has shown that chronic stress (associated with depression) is also linked to glucocorticoid receptor resistance, as well as problems with the regulation of the inflammatory response [484]. Further work is needed on this and the underpinning mechanisms between the determinants and outcomes. This review highlighted the myriad ways of measuring depression and its determinants [66,85,281,298,451,485]. Thus, the standardization of the measurements of the outcomes (ex. a gold standard for measuring depression) and determinants is essential; this can facilitate comparisons of findings across studies.

### 5.1. Strengths

This paper has important strengths. It brings together the wide literature on depression and helps to bridge disciplines in relation to one of the most common mental health problems. We identified, selected, and extracted data from studies, and provided concise summaries.

### 5.2. Limitations

The limitations of the review include missing potentially important studies; however, this is a weakness that cannot be avoided by literature reviews. Nevertheless, the aim of the review was not to identify each study that has been conducted on the risk and protective factors of depression (which a single review is unable to capture) but rather to gain insight into the breadth of literature on this topic, highlight key biological, psychological, and social determinants, and shed light on important themes, such as the lifecourse perspective and gene–environment interactions.

## 6. Conclusions

We have reviewed the determinants of depression and recognize that there are a multitude of risk and protective factors at the individual and wider ecologic levels. These determinants are interlinked and influence one another. We have attempted to describe the wide literature on this topic, and we have brought to light major factors that are of public mental health significance. This review may be used as an evidence base by those in public health, clinical practice, and research.

This paper discusses key areas in depression research; however, an exhaustive discussion of all the risk factors and determinants linked to depression and their mechanisms is not possible in one journal article—which, by its very nature, a single paper cannot do. We have brought to light overarching factors linked to depression and a workable conceptual framework that may guide clinical and public health practice; however, we encourage other researchers to continue to expand on this timely and relevant work—particularly as depression is a top priority on the policy agenda now.

## Supplementary Materials

The following are available online at https://www.mdpi.com/article/10.3390/brainsci11121633/s1, Figure S1: Conceptual framework: Determinants of depression, Table S1: Data charting—A selection of determinants from the literature.

## Appendix A

### Appendix A.1. Search Strategy

#### PubMed

Search: ((((((((((((((((“Gene-Environment Interaction”[Majr]) OR (“Genetics”[Mesh])) OR (“Genome-Wide Association Study”[Majr])) OR (“Microbiota”[Mesh] OR “Gastrointestinal Microbiome”[Mesh])) OR (“Neurogenic Inflammation”[Mesh])) OR (“genetic determinant”)) OR (“gut-brain-axis”)) OR (“Kynurenine”[Majr])) OR (“Cognition”[Mesh])) OR (“Neuronal Plasticity”[Majr])) OR (“Neurogenesis”[Mesh])) OR (“Genes”[Mesh])) OR (“Neurology”[Majr])) OR (“Social Determinants of Health”[Majr])) OR (“Glucocorticoids”[Mesh])) OR (“Tryptophan”[Mesh])) AND (“Depression”[Mesh] OR “Depressive Disorder”[Mesh]) Filters: from 2017—2020.

Ovid MEDLINE(R) and Epub Ahead of Print, In-Process, In-Data-Review & Other Non-Indexed Citations, Daily and Versions(R)

1. exp *Depression/
2. exp *Depressive Disorder/
3. 1 or 2
4. exp *”Social Determinants of Health”/
5. exp *Tryptophan/
6. exp *Glucocorticoids/
7. exp *Neurology/
8. exp *Genes/
9. exp *Neurogenesis/
10. exp *Neuronal Plasticity/
11. exp *Kynurenine/
12. exp *Genetics/
13. exp *Neurogenic Inflammation/
14. exp *Gastrointestinal Microbiome/
15. exp *Genome-Wide Association Study/
16. exp *Gene-Environment Interaction/
17. exp *Depression/et [Etiology]
18. exp *Depressive Disorder/et
19. 17 or 18
20. or/4-16   637368
21. 3 and 20
22. 19 or 21
23. limit 22 to yr = “2017–Current”
24. “cause* of depression”.mp.
25. “cause* of depression”.ti.
26. 23 or 25
27. (cause adj3 (depression or depressive)).ti.
28. (caus* adj3 (depression or depressive)).ti.
29. 23 or 28

### Appendix A.2. PsycInfo

#	**Query**	**Limiters/Expanders**	**Last Run Via**
S10	(S3 OR S4 OR S5 OR S6 OR S7) AND (S1 OR S8)	Limiters-Publication Year: 2017–2021 Expanders-Apply equivalent subjects Search modes-Boolean/Phrase	Interface-EBSCOhost Research Databases Search Screen-Advanced Search Database-APA PsycInfo
S9	(S3 OR S4 OR S5 OR S6 OR S7) AND (S1 OR S8)	Expanders-Apply equivalent subjects Search modes-Boolean/Phrase	Interface-EBSCOhost Research Databases Search Screen-Advanced Search Database-APA PsycInfo
S8	S3 OR S4 OR S5 OR S6 OR S7	Expanders-Apply equivalent subjects Search modes-Boolean/Phrase	Interface-EBSCOhost Research Databases Search Screen-Advanced Search Database-APA PsycInfo
S7	TI (Social Determinants of Health)	Expanders-Apply equivalent subjects Search modes-Boolean/Phrase	Interface-EBSCOhost Research Databases Search Screen-Advanced Search Database-APA PsycInfo
S6	TI (Neurogenic Inflammation)	Expanders-Apply equivalent subjects Search modes-Boolean/Phrase	Interface-EBSCOhost Research Databases Search Screen-Advanced Search Database-APA PsycInfo
S5	TI (Genome-Wide Association Study)	Expanders-Apply equivalent subjects Search modes-Boolean/Phrase	Interface-EBSCOhost Research Databases Search Screen-Advanced Search Database-APA PsycInfo
S4	TI (Gene-Environment Interaction)	Expanders-Apply equivalent subjects Search modes-Boolean/Phrase	Interface-EBSCOhost Research Databases Search Screen-Advanced Search Database-APA PsycInfo
S3	(((((MM “Etiology” OR MM “Causality”) OR (MM “Tryptophan” OR MM “Hydroxytryptophan (5-)”)) OR (MM “Glucocorticoids” OR MM “Dexamethasone”)) OR (MM “Neurology”)) AND (MM “Genes” OR MM “Alleles” OR MM “CLOCK Gene” OR MM “Immediate Early Genes” OR MM “Quantitative Trait Loci” OR MM “Genetics” OR MM “Behavioral Genetics” OR MM “Epigenetics” OR MM “Eugenics” OR MM “Genetic Engineering” OR MM “Genetic Processes” OR MM “Genomics” OR MM “Optogenetics” OR MM “Pharmacogenetics” OR MM “Population Genetics”)) OR (MM “Gastrointestinal Microbiota”)	Expanders-Apply equivalent subjects Search modes-Boolean/Phrase	Interface-EBSCOhost Research Databases Search Screen-Advanced Search Database-APA PsycInfo
S2	MM “Major Depression” OR MM “Anaclitic Depression” OR MM “Dysthymic Disorder” OR MM “Endogenous Depression” OR MM “Late Life Depression” OR MM “Postpartum Depression” OR MM “Reactive Depression” OR MM “Recurrent Depression” OR MM “Treatment Resistant Depression”	Expanders-Apply equivalent subjects Search modes-Boolean/Phrase	Interface-EBSCOhost Research Databases Search Screen-Advanced Search Database-APA PsycInfo
S1	TI caus* n3 (depression or depressive)	Expanders-Apply equivalent subjects Search modes-Boolean/Phrase	Interface-EBSCOhost Research Databases Search Screen-Advanced Search Database-APA PsycInfo

#### Scopus

(TITLE (*depression* OR “*Depressive Disorder*”) AND TITLE (“*Social Determinants of Health*” OR *tryptophan* OR *glucocorticoids* OR *neurology* OR *genes* OR *neurogenesis* OR “*Neuronal Plasticity*” OR *kynurenine* OR *genetics* OR “*Neurogenic Inflammation*” OR “*Gastrointestinal Microbiome*” OR “*Genome-Wide Association Study*” OR “*Gene-Environment Interaction*” OR *aetiology* OR *etiology*)) OR TITLE (*cause** W/3 (*depression* OR *depressive*)).
