# Supplementary material for: Biological, Psychological, and Social Determinants of Depression: A Review of Recent Literature
Source: Brain Sci. 2021 Dec 10;11(12):1633. doi: 10.3390/brainsci11121633 (PMC8699555; doi:10.3390/brainsci11121633)
Supplement: Supplementary file 1 [file brainsci-11-01633-s001.zip › Supplementary Table S1.pdf]

Table S1. Data charting table – a selection of determinants from the literature

Each study contains a selection of determinants; related outcomes are provided.

Last name of first author and year	Title	Determinant	Outcome
Pullmer 2020	Cognitive and behavioral correlates of depressive symptoms in a community sample of adolescents	obsessive-compulsive symptoms, thought-action fusion, thought-shape fusion, and eating pathology	depressive symptoms
Houtepen 2020	Associations of adverse childhood experiences with educational attainment and adolescent health and the role of family and socioeconomic factors: A prospective cohort study in the UK	Adverse childhood experiences	Depression
Firth 2020	Food and mood: how do diet and nutrition affect mental wellbeing?	Food	Depression
Bone 2020	The role of gender inequalities in adolescent depression	Gender inequality	Depression

Arnetz 2020	Dysfunctional neuroplasticity in newly arrived Middle Eastern refugees in the U.S.: Association with environmental exposures and mental health symptoms	Neurotrophins combined into a neuroplasticity index	Depression
Zhou 2019	Who has low health literacy and does it matter for depression? Findings from aggregated and disaggregated racial/ethnic groups	Health literacy	Depression
Zhao 2019	Neuroimmune Advance in Depressive Disorder	stress response, neuroendocrine, neurotransmission, neurogenesis, and gut microbiota, (neuro)inflammation	Major depressive disorder
Zhang 2019	Association between fatigue and depressive symptoms among kidney transplantation recipients: The mediating role of rumination	Fatigue	depressive symptoms
Yamanishi 2019	Interleukin-18-deficient mice develop hippocampal abnormalities related to possible depressive-like behaviors	IL-18 and death of hippocampal cells	depressive-like behaviour

Ward 2019	Relational impairments, sluggish cognitive tempo, and severe inattention are associated with elevated self-rated depressive symptoms in adolescents with ADHD	higher SCT symptom severity, lower hyperactive/impulsive (HI) symptom severity, higher social impairments, higher conflict with parents, and lower academic problems	depressive symptoms
Wang 2019	High urea induces depression and LTP impairment through mTOR signalling suppression caused by carbamylation	urea accumulation in brain	Depression
Wang 2019	Fr-HMGB1 and ds-HMGB1 activate the kynurenine pathway via different mechanisms in association with depressive-like behavior	kynurenine pathway, different redox states of HMGB1	Depression
Van Dalfsen 2019	The involvement of sleep in the relationship between the serotonin transporter gene-linked polymorphic region (5-HTTLPR) and depression: A systematic review	serotonin transporter gene-linked polymorphic region (5-HTTLPR)	(Stress-related) depression

Valles-Colomer 2019	The neuroactive potential of the human gut microbiota in quality of life and depression	human gut microbiota	Depression
Tsai 2019	Ambivalence over emotional expression and intrusive thoughts as moderators of the link between self-stigma and depressive symptoms among Chinese American breast cancer survivors	Self-stigma	Depression
Trick 2019	Perseverative negative thinking predicts depression in people with acute coronary syndrome	Perseverative negative thinking	Depression
Torres-Berrío 2019	Unraveling the epigenetic landscape of depression: focus on early life stress	Early life stress, epigenetics	Depression
Teshigawara 2019	Changes in tryptophan metabolism during pregnancy and postpartum periods: Potential involvement in postpartum depressive symptoms	Tryptophan (TRP) metabolism, plasma levels of KYN and KA, and KYN/TRP and KA/KYN ratio, plasma level of 3HAA	Postpartum depression
Stower 2019	Depression linked to the microbiome	Microbiome and metabolic capabilities	Depression

Speed 2019	Investigating the causal relationship between neuroticism and depression via Mendelian randomization	Neuroticism	Depression
Song 2019	Metabolomic signatures and microbial community profiling of depressive rat model induced by adrenocorticotrophic hormone	gut microbiota	depression
Smeeth 2019	Polygenic risk for circulating reproductive hormone levels and their influence on hippocampal volume and depression susceptibility	Reproductive hormones	Major depressive disorder
Simkin 2019	Microbiome and Mental Health, Specifically as It Relates to Adolescents	microbiome, the gut-brain axis	depression
Saul 2019	Polymorphism in the serotonin transporter gene polymorphisms (5-HTTLPR) modifies the association between significant life events and depression in people with multiple sclerosis	Significant life events	Depression

Sanders 2019	A role for the microbiome in mother-infant interaction and perinatal depression	maternal microbiota	Depression
Patel 2019	Rodent models of social stress and neuronal plasticity: Relevance to depressive-like disorders	Social stress (rodents)	Depression
Park 2019	Significant role of gene-gene interactions of clock genes in mood disorder	Genes	Mood disorder (major depressive disorder and bipolar disorder)
Orchard 2019	I Am Worthless and Kind'; the specificity of positive and negative self-evaluation in adolescent depression	Self-concept	Depression
Normann 2019	Gene-environment interactions between HPA-axis genes and stressful life events in depression: a systematic review	HPA-axis genes and stressful life events	Depression
Nissen 2019	Low-level cognitive ability in young adulthood and other risk factors of depression in an observational cohort study among deployed Danish soldiers	cognitive ability	Depression

Nankoo 2019	Examining the Rate of Self-Reported ADHD-Related Traits and Endorsement of Depression, Anxiety, Stress, and Autistic-Like Traits in Australian University Students	ADHD-related symptoms	Depression
Mutyambizi 2019	Subjective social status and inequalities in depressive symptoms: a gender-specific decomposition analysis for South Africa	Inequalities, social status	Depression
Moulton 2019	Depressive symptoms in inflammatory bowel disease: an extraintestinal manifestation of inflammation?	inflammation and dysregulation of the gut-brain axis	depressive symptoms
Milenkovic 2019	The Role of Chemokines in the Pathophysiology of Major Depressive Disorder	chemokines	Major depressive disorder
Metel 2019	Resilience and cognitive biases mediate the relationship between early exposure to traumatic life events and depressive symptoms in young adults	early exposure to traumatic life events	depressive symptoms

Ma 2019	Chronic paradoxical sleep deprivation-induced depression-like behavior, energy metabolism and microbial changes in rats	circadian disturbance, chronic PSD-induced alterations in gut microbiota and related host changes in metabolism	Depression-like behaviour
Lv 2019	The Gut Microbiome Modulates the Changes in Liver Metabolism and in Inflammatory Processes in the Brain of Chronic Unpredictable Mild Stress Rats	dysbiosis gut microbiome	Depression
Lu 2019	Community-based structural social capital and depressive symptoms of older urban Chinese adults: The mediating role of cognitive social capital	Structural social capital	depressive symptoms
Liu 2019	Prioritization and comprehensive analysis of genes related to major depressive disorder	genes	Major depressive disorder
Liu 2019	A Latent Structure Analysis of Cognitive Vulnerability to Depression in Adolescence	Cognitive vulnerability	Depression

Liang 2019	Exploring causal pathways linking cerebral small vessel diseases burden to poststroke depressive symptoms with structural equation model analysis	Cerebral small vessel diseases (SVD)	Poststroke depressive symptoms
Li 2019	Integrative analyses of major histocompatibility complex loci in the genome-wide association studies of major depressive disorder	Genes	Depression
Kuo 2019	Moody microbiome: Challenges and chances	Gut microbiota	Depression
Ksinan 2019	Genetic and environmental effects on the development of depressive symptoms from adolescence to adulthood in a nationally representative sample	Genetic and environmental effects	depressive symptoms
Kruse 2019	Kynurenine metabolism and inflammation-induced depressed mood: A human experimental study	Kynurenine pathway	Depressed mood

Konsztowicz 2019	The role of illness engulfment in the association between insight and depressive symptomatology in schizophrenia	Insight	depressive symptoms
Kong 2019	The association of endocannabinoid receptor genes (CNR1 and CNR2) polymorphisms with depression: A meta-analysis	Gene variants	Depression susceptibility
Kojima 2019	BDNF pro-peptide: physiological mechanisms and implications for depression	BDNF pro-peptide	Major depressive disorder
Khaled 2019	Prevalence and potential determinants of subthreshold and major depression in the general population of Qatar	Ethnicity, migrant status	Major depressive disorder and subthreshold disorder
Kelly 2019	Mood and Microbes: Gut to Brain Communication in Depression	Gut microbiota	Depression
Kahl 2019	Link between depression and cardiovascular diseases due to epigenomics and proteomics: Focus on energy metabolism	Cardiovascular disease; lifestyle factors (e.g. physical activity, smoking behavior), dysfunctions of endocrine systems (e.g. hypothalamus-pituitary adrenal axis), and a dysbalance of pro- and anti-inflammatory factors	Depression

Jianguo 2019	Altered gut metabolome contributes to depression-like behaviors in rats exposed to chronic unpredictable mild stress	gut metabolome, stress	Depression-like behaviour
Jia 2019	Ciliary neurotrophic factor is a key sex-specific regulator of depressive-like behavior in mice	Ciliary neurotrophic factor (CNTF)	Depressive-like behaviour
Jacobson 2019	Glucocorticoid receptor deletion from locus coeruleus norepinephrine neurons promotes depression-like social withdrawal in female but not male mice	Glucocorticoid receptor deletion	Depression-like social withdrawal
Inserra 2019	Neuroimmunomodulation in Major Depressive Disorder: Focus on Caspase 1, Inducible Nitric Oxide Synthase, and Interferon-Gamma	Inflammation, stress	Major depressive disorder
Innes 2019	Microglial-driven changes in synaptic plasticity: A possible role in major depressive disorder	Microglia	Major depressive disorder

Hufner 2019	How acute and chronic physical disease may influence mental health - An Analysis of neurotransmitter precursor amino acid levels	inflammatory reactions, PHE/TYR	Mental health- Depression
Hu 2019	Inflammation-dependent ISG15 upregulation mediates MIA-induced dendrite damages and depression by disrupting NEDD4/Rap2A signaling	* Maternal immune activation	depression-like behaviors
Holmes 2019	Aberrant Epigenomic Modulation of Glucocorticoid Receptor Gene (NR3C1) in Early Life Stress and Major Depressive Disorder Correlation: Systematic Review and Quantitative Evidence Synthesis	Early life stress	Major depressive disorder
Hei 2019	Effects of chronic mild stress induced depression on synaptic plasticity in mouse hippocampus	Chronic mild stress	Depression-like behaviours

Halahakoon 2019	Cognitive Impairment and Depression—Cause, Consequence, or Coincidence?	Cognitive impairment	Depression
Gong 2019	Proteomic analysis of the intestine reveals SNARE-mediated immunoregulatory and amino acid absorption perturbations in a rat model of depression	gut-brain axis	Depression
Gerring 2019	A gene co-expression network-based analysis of multiple brain tissues reveals novel genes and molecular pathways underlying major depression	genes and gene co-expression networks	Major depression
Gabrys 2019	Self-Reported Mild Traumatic Brain Injuries in Relation to Rumination and Depressive Symptoms: Moderating Role of Sex Differences and a Brain-Derived Neurotrophic Factor Gene Polymorphism	Mild traumatic brain injuries	depressive symptoms (and rumination)

Frangou 2019	Insulin resistance: Genetic associations with depression and cognition in population based cohorts	Insulin resistance traits	Depression
Fossati 2019	Circuit based anti-correlation, attention orienting, and major depression	(Neural networks), social rejection	Depression
Fang 2019	Do daily dynamics in rumination and affect predict depressive symptoms and trait rumination? An experience sampling study	Entropy	Depressive symptoms
Fabbri 2019	The Genetics of Treatment-Resistant Depression: A Critical Review and Future Perspectives	Variants in genes	treatment-resistant depression
Elwood 2019	A systematic review investigating if genetic or epigenetic markers are associated with postnatal depression	interaction between epigenetic, genetic, hormonal and environmental factors (ex. genetic polymorphisms, stressful life events, childhood adversity)	Postnatal depression

Duan 2019	The IDO genetic polymorphisms and postpartum depressive symptoms: an association study in Chinese parturients who underwent cesarean section	Genetic polymorphism, kynurenine pathway	Postpartum depressive symptoms
Druzhkova 2019	Acute stress response to a cognitive task in patients with major depressive disorder: potential metabolic and proinflammatory biomarkers	stress response	Major depressive disorder
DeJong 2019	Does rumination mediate the relationship between attentional control and symptoms of depression?	Attentional control	Depression
Dam 2019	Clinical association to FKBP5 rs1360780 in patients with depression	genes	Unipolar depression
Chung 2019	Exploration of microbiota targets for major depressive disorder and mood related traits	Microbiota	Major depressive disorder

Chen 2019	The effects of glucocorticoids on depressive and anxiety-like behaviors, mineralocorticoid receptor-dependent cell proliferation regulates anxiety-like behaviors	chronic unpredictable stress	Depressive-like behaviour
Chen 2019	Hippocampal Genetic Knockdown of PPARδ Causes Depression-Like Behaviors and Neurogenesis Suppression	PPARδ	Depressive-like behaviours
Carvalho 2019	Cognitive fusion and depressive symptoms in women with chronic pain: A longitudinal growth curve modelling study over 12 months	Cognitive fusion	depressive symptoms
Cadden 2018	Cognitive Reserve Attenuates the Effect of Disability on Depression in Multiple Sclerosis	Disability	Depression
Border 2019	No Support for Historical Candidate Gene or Candidate Gene-by-Interaction Hypotheses for Major Depression Across Multiple Large Samples	Genes	a number of operational definitions of depression used (e.g., lifetime diagnosis, current severity, episode recurrence)

Blote 2019	The temporal association between emotional clarity and depression symptoms in adolescents	emotional clarity	Depression
Bernstein 2019	Unique and predictive relationships between components of cognitive vulnerability and symptoms of depression	cognitive vulnerability / style, attributions	depressive symptoms
Barone 2019	The 'Yin' and the 'Yang' of the kynurenine pathway: excitotoxicity and neuroprotection imbalance in stress-induced disorders	tryptophan/catabolites (TRY/CAT) pathway, inflammation	Major depressive disorder
Bailen 2019	Meta-emotions in daily life: Associations with emotional awareness and depression	Meta-emotional experience	Depression
Aldoghachi 2019	Screening of brain-derived neurotrophic factor (BDNF) single nucleotide polymorphisms and plasma BDNF levels among Malaysian major depressive disorder patients	BDNF allele	Major depressive disorder

Agnihotri 2019	PINK1 deficiency is associated with increased deficits of adult hippocampal neurogenesis and lowers the threshold for stress-induced depression in mice	stress	Stress-induced depression
Zlatar 2018	Subjective Cognitive Decline Correlates With Depression Symptoms and Not With Concurrent Objective Cognition in a Clinic-Based Sample of Older Adults	subjective cognitive decline	depression
Zhao 2018	BDNF Val66Met polymorphism, life stress and depression: A meta-analysis of gene-environment interaction	Stressful life events, childhood adversity	Depression
Zhang 2018	CDK5-mediated phosphorylation of Sirt2 contributes to depressive-like behavior induced by social defeat stress	Histone deacetylases	Depression, depressive-like behaviour

Zhang 2018	Dvl3 polymorphism interacts with life events and pro-inflammatory cytokines to influence major depressive disorder susceptibility	Dvl3 allelic variations, negative life events, pro-inflammatory cytokines	Major depressive disorder
Zhang 2018	Neuroimaging genomic studies in major depressive disorder: A systematic review	Genes	Major depressive disorder
Zang 2018	Regulation of proinflammatory monocyte activation by the kynurenine-AhR axis underlies immunometabolic control of depressive behavior in mice	Kynurenine, inflammatory signalling	Depression
Zamora-Racaza 2018	Role of psychosocial reserve capacity in anxiety and depression in patients with systemic lupus erythematosus	Low self-esteem, optimism, coping and social support	Depression
Zalar 2018	The Role of Microbiota in Depression - a brief review	Microbiota	Depressive disorders
Youssef 2018	Association of BDNF Val66Met Polymorphism and Brain BDNF Levels with Major Depression and Suicide	brain brain-derived neurotrophic factor and the brain-derived neurotrophic factor Met allele	Depression

Yang 2018	Quantitative proteomic study of the plasma reveals acute phase response and LXR/RXR and FXR/RXR activation in the chronic unpredictable mild stress mouse model of depression	chronic unpredictable mild stress, (proteins)	Depression
Xiao 2018	The Gene Encoding Protocadherin 9 (PCDH9), a Novel Risk Factor for Major Depressive Disorder	Genes	Major depressive disorder
Wu 2018	PI3K/AKT/GSK3β/CRMP-2-mediated neuroplasticity in depression induced by stress	Early stress, neuroinflammatory factors	Depression
Winter 2018	Gut microbiome and depression: what we know and what we need to know	Gut microbiome	Depression
Wigner 2018	Variation of genes encoding KAT1, AADAT and IDO1 as a potential risk of depression development	Genes involved in TRYCATs pathway	Depressive disorder
Wigner 2018	Association between single nucleotide polymorphisms of TPH1 and TPH2 genes, and depressive disorders	Genes, polymorphisms	Depressive disorder

Wigner 2018	Variation of genes involved in oxidative and nitrosative stresses in depression	Genes	Depression
Wigner 2018	The molecular aspects of oxidative & nitrosative stress and the tryptophan catabolites pathway (TRYCATs) as potential causes of depression	TRYCAT pathway, antioxidants, nitric oxide synthase	Depression
Wang 2018	Interaction between early-life stress and FKBP5 gene variants in major depressive disorder and post-traumatic stress disorder: A systematic review and meta-analysis	Genotype, early life stress	Major depression
Wang 2018	Association study of NDST3 gene for schizophrenia, bipolar disorder, major depressive disorder in the Han Chinese population	Gene	Depressive disorder
Uchida 2017	Epigenetic mechanisms of major depression: Targeting neuronal plasticity	Genes, non-genetic factors (ex. stressful life events)	Depression

Trindade 2018	Chronic illness-related cognitive fusion explains the impact of body dissatisfaction and shame on depression symptoms in breast cancer patients	body image dissatisfaction and chronic illness shame	depressive symptoms
Tozzi 2018	Epigenetic Changes of FKBP5 as a Link Connecting Genetic and Environmental Risk Factors with Structural and Functional Brain Changes in Major Depression	Genes, childhood maltreatment	Major depressive disorder
Smith 2018	Cognitive biases predict symptoms of depression, anxiety and wellbeing above and beyond neuroticism in adolescence	Rumination, dysfunctional attitudes, and negative biases in ambiguous scenarios interpretation and self-referential memory, neuroticism	Depression
Shimada 2018	An epigenome-wide methylation study of healthy individuals with or without depressive symptoms	DNA methylation	Depression
Senra 2018	Exploring the link between perfectionism and depressive symptoms: Contribution of rumination and defense styles	maladaptive perfectionism	Depression

Schachter 2018	Effects of obesity on depression: A role for inflammation and the gut microbiota	obesity	depression
Rantanen 2018	Awareness of hypertension and depressive symptoms: a cross-sectional study in a primary care population	gender, alcohol use, smoking status, physical activity	depressive symptoms
Quinn 2018	Negative cognitive style and cortisol recovery accentuate the relationship between life stress and depressive symptoms	Negative cognitive style, cortisol recovery, stressful life events	Symptoms of depression
Prussien 2018	Cognitive Function, Coping, and Depressive Symptoms in Children and Adolescents with Sickle Cell Disease	Cognitive function, coping	depressive symptoms
Porras-Segovia 2018	Prevalence and correlates of major depression in Granada, Spain: Results from the GranadΣp study	Gender, family history of mental illness, neuroticism, number of life threatening events (LTE), physical health status, cognitive impairment and cannabis use	Major depressive disorder
Perin 2018	Amyloid burden and incident depressive symptoms in preclinical Alzheimer's disease	amyloid burden	Depressive symptoms

Park 2018	Trauma and Depression among North Korean Refugees: The Mediating Effect of Negative Cognition	early traumatic experiences	depressive symptoms
Otani 2018	Interrelations among negative core beliefs, attachment anxiety and low self-directedness, putative central constructs of depression vulnerabilities in cognitive, attachment and psychobiological personality theories	Negative self-image	Depression vulnerability
Orchard 2018	The combined influence of cognitions in adolescent depression: Biases of interpretation, self-evaluation, and memory	cognitive biases	depression
Noda 2018	Impaired neuroplasticity in the prefrontal cortex in depression indexed through paired associative stimulation	neuroplasticity	depression

Naoi 2018	Type A monoamine oxidase and serotonin are coordinately involved in depressive disorders: from neurotransmitter imbalance to impaired neurogenesis	MAOA, serotonin, gene-environment interactions	Depressive behaviours
Nagy 2018	A role for activity-dependent epigenetics in the development and treatment of major depressive disorder	stress, epigenetic factors	major depressive disorder
Mundorf 2018	Methylation of MORC1: A possible biomarker for depression?	gene	major depression
Mekli 2018	Genome-wide scan of depressive symptomatology in two representative cohorts in the United States and the United Kingdom	Genes	Depression
McCormick 2018	Socioeconomic Predictors of Incident Depression in Systemic Lupus Erythematosus	socioeconomic predictors	Depression

Mac Giollabhui 2018	Negative cognitive style interacts with negative life events to predict first onset of a major depressive episode in adolescence via hopelessness	hopelessness, (negative inferential style)	depression
Liu 2018	Beta-defensin 1, aryl hydrocarbon receptor and plasma kynurenine in major depressive disorder: metabolomics-informed genomics	Kynurenine, genes	Major depressive disorder
Lima-Ojeda 2018	Neurobiology of depression: A neurodevelopmental approach	genes, (early stress), BDNF, HPA axis, brain abnormalities	depression
Lam 2018	DNA methylation and genetic variation of the angiotensin converting enzyme (ACE) in depression	ACE methylation	depression
Lam 2018	Genotype-dependent associations between serotonin transporter gene (SLC6A4) DNA methylation and late-life depression	methylation	late-life depression

Kushner 2018	Depression in early adolescence: Contributions from relational aggression and variation in the oxytocin receptor gene	genes, interpersonal risk factors	depression
Kube 2018	Integrating situation-specific dysfunctional expectations and dispositional optimism into the cognitive model of depression - A path-analytic approach	Dysfunctional expectations	Major depression
Kube 2018	Situational expectations mediate the effect of global beliefs on depressive symptoms: a 1-year prospective study using a student sample	global beliefs (such as intermediate beliefs and dispositional optimism)	depressive symptoms
Kitzlerová 2018	Interactions Among Polymorphisms of Susceptibility Loci for Alzheimer's Disease or Depressive Disorder	polymorphisms	Major depression
Kang 2018	Longitudinal associations between glucocorticoid receptor methylation and late-life depression	methylation of genes	late-life depression

Jesulola 2018	Understanding the pathophysiology of depression: From monoamines to the neurogenesis hypothesis model - are we there yet?	neurotransmitter, genetic, environmental, immunologic, endocrine factors, and neurogenesis, HPA axis	depression
Huang 2018	Neural circuitry among connecting the hippocampus, prefrontal cortex and basolateral amygdala in a mouse depression model: Associations correlations between BDNF levels and BOLD - fMRI signals	(BDNF), chronic unpredictable mild stress	Depression-like behaviour
Hu 2018	No association of GRIN2A polymorphisms with the major depressive disorder in the Chinese Han origin	polymorphisms (genetics)	Major depressive disorder
Hing 2018	A comprehensive review of genetic and epigenetic mechanisms that regulate BDNF expression and function with relevance to major depressive disorder	BDNF, stress	MDD

He 2018	Interaction between job stress and the BDNF Val66Met polymorphism affects depressive symptoms in Chinese healthcare workers	Job-related stress, BDNF polymorphism	Depression
Haverkamp 2018	Differences in the association of inflammation and tryptophan with depressive symptoms between white and non-white chronic dialysis patients	Inflammation, tryptophan	Depressive symptoms
Hankin 2018	Cognitive and Interpersonal Vulnerabilities to Adolescent Depression: Classification of Risk Profiles for a Personalized Prevention Approach	cognitive vulnerability, interpersonal support, and interpersonal conflict	Depression
Hallgren 2018	Passive and mentally-active sedentary behaviors and incident major depressive disorder: A 13-year cohort study	passive (e.g. watching TV) and mentally-active (e.g. office-work) sedentary behaviour	Depression

Guida 2018	Antibiotic-induced microbiota perturbation causes gut endocannabinoidome changes, hippocampal neuroglial reorganization and depression in mice	Microbiome, antibiotic	Depression
Geisler 2018	Immunometabolism in the Pathogenesis of Depressive Disorders - Therapeutic Considerations	genetics, environment, lifestyle, immunological status	Depression
Gariballa 2018	Association between muscle function, cognitive state, depression symptoms and quality of life of older people: evidence from clinical practice	low muscle function/strength	Depression symptoms
Garcia 2018	Brooding as moderator of depressive symptoms after a work accident: A longitudinal study	Work accident	depressive symptoms
Enko 2018	Assessment of tryptophan metabolism and signs of depression in individuals with carbohydrate malabsorption	lactose malabsorption, fructose malabsorption, TRP metabolites	depression

Duan 2018	The role of tryptophan metabolism in postpartum depression	tryptophan metabolism, kynurenine pathway products	post-partum depression
Denis 2018	Cognitive factors and post-partum depression: What is the influence of general personality traits, rumination, maternal self-esteem, and alexithymia?	cognitive factors (neuroticism, brooding rumination, and low maternal self-esteem, alexithymia)	post-partum depression
da Conceição 2018	Implication of galanin gene rs948854 polymorphism in depressive symptoms in adolescents	alleles, genotypes	depressive symptoms
Christensen 2018	Young adult cognitive ability and subsequent major depression in a cohort of 666,804 Danish men	young adult cognitive ability	major depression
Burke 2018	Latent classes of trait affect and cognitive affective regulation strategies are associated with depression, non-suicidal self-injury, and well-being	Coping	Depressive symptoms

Brouillard 2018	Links Between the Mother-Adolescent and Father-Adolescent Relationships and Adolescent Depression: A Genetically Informed Study	Support and conflict, genetic vulnerability	depressive symptoms
Bondar 2018	Molecular Adaptations to Social Defeat Stress and Induced Depression in Mice	social defeat stress, (genes)	depression
Boku 2018	Neural basis of major depressive disorder: Beyond monoamine hypothesis	hippocampus, HPA axis, glucocorticoids, (monoamine)	Major depressive disorder
Bleys 2018	Gene-environment interactions between stress and 5-HTTLPR in depression: A meta-analytic update	stress, 5-HTTLPR	depression
Backhouse 2018	Cognitive ability, education and socioeconomic status in childhood and risk of post-stroke depression in later life: A systematic review and meta-analysis	education	post-stroke depression

Ashokan 2018	Housing environment influences stress-related hippocampal substrates and depression-like behavior	Impoverished/ enriched sensory environments; stress and sensory enrichment	Depressive-like behaviour
Arcego 2018	Impact of High-Fat Diet and Early Stress on Depressive-Like Behavior and Hippocampal Plasticity in Adult Male Rats	High fat diet, early stress (social isolation)	Depressive-like behaviour
Anderson 2018	Linking the biological underpinnings of depression: Role of mitochondria interactions with melatonin, inflammation, sirtuins, tryptophan catabolites, DNA repair and oxidative and nitrosative stress, with consequences for classification and cognition	immune-inflammation, tryptophan catabolites (TRYCATs), mitochondrial dysfunction, aryl hydrocarbonn receptor activation, and oxidative and nitrosative stress (O&NS), as well as decreased sirtuins and melatonergic pathway activity, prenatal stress, gut-brain axis	Major depressive disorder
Zhou 2017	Life events and hopelessness depression: The influence of affective experience	Affective experience (of life events); negative, positive, neutral life events, negative cognitive style	Hopelessness depression

Zhang 2017	Interaction of estrogen receptor β and negative life events in susceptibility to major depressive disorder in a Chinese Han female population	Estrogen receptor β (ESR2), (negative life events)	Major depressive disorder
Yohn 2017	The role of 5-HT receptors in depression	serotonin	depression
Yang 2017	Lucky gene 5-HTTLPR and postpartum depression: A systematic review	gene 5-HTTLPR	Postpartum depression
Yang 2017	Bifidobacterium in the gut microbiota confer resilience to chronic social defeat stress in mice	gut microbiota	depression
Yang 2017	The relationship between cognition and depressive symptoms, and factors modifying this association, in Alzheimer's disease: A multivariate multilevel model	cognition , family status, employment before retirement, homemaking, reading, aluminum utensil use, dietary restriction, and hypertension	depression

Xu 2017	The interaction of miR-34b/c polymorphisms and negative life events increases susceptibility to major depressive disorder in Han Chinese population	miR-34b/c polymorphisms, (negative life events)	major depressive disorder
Wigner 2017	Oxidative and Nitrosative Stress as Well as the Tryptophan Catabolites Pathway in Depressive Disorders	oxidative and nitrosative stress, tryptophan catabolites pathway, nitric oxide synthase, genetic factors, monoaminergic factors, hormonal regulation disorders, biological rhythm sleep-wake, neuropeptides, immune factors, structural changes in central nervous sytem, various diseases and medications	depressive disorders
Vermeer 2017	Correlates of depressive symptoms in individuals attending outpatient stroke clinics	cognitive deficits, pain, tobacco use, being enrolled in therapy	depressive symptoms
Van Assche 2017	Gene-based interaction analysis shows GABAergic genes interacting with parenting in adolescent depressive symptoms	perceived parental support, psychological control, and harsh punishment, genes	depressive symptoms

Trindade 2017	An 18-month study of the effects of IBD symptomatology and emotion regulation on depressed mood	IBD, emotion regulation processes (brooding, cognitive fusion, rumination)	Depressed mood
Treutlein 2017	Association between neuropeptide Y receptor Y2 promoter variant rs6857715 and major depressive disorder	NPY2R rs6857715 C-allele	major depressive disorder
Swartz 2017	An epigenetic mechanism links socioeconomic status to changes in depression-related brain function in high-risk adolescents	SES, (HPA axis, methylation, amygdala) risk-related brain function,	depressive symptoms
Strasser 2017	Mechanisms of Inflammation-Associated Depression: Immune Influences on Tryptophan and Phenylalanine Metabolisms	immune system, tryptophan metabolism, tryptophan metabolism, IDO-1	depression
Strachan 2017	Neighborhood deprivation and depression in adult twins: genetics and gene×environment interaction	Neighbourhood deprivation, (genes)	Depression

Stange 2017	Perseverate or decenter? Differential effects of metacognition on the relationship between parasympathetic inflexibility and symptoms of depression in a multi-wave study	parasympathetic inflexibility	Symptoms of depression
Slykerman 2017	Antibiotics in the first year of life and subsequent neurocognitive outcomes	antibiotics	depression symptoms
Shi 2017	Methylation Status of the Serotonin Transporter Promoter CpG Island Is Associated With Major Depressive Disorder in Chinese Han Population: A Case-Control Study	serotonin transporter (5-HTT), education	major depressive disorder
Serafini 2017	Abnormalities in Kynurenine Pathway Metabolism in Treatment-Resistant Depression and Suicidality: A Systematic Review	Kynurenine pathway, neuroinflammation	treatment-resistant depression

Savitz 2017	Role of Kynurenine Metabolism Pathway Activation in Major Depressive Disorders	kynurenine pathway, neurotoxic and neuroprotective metabolites	depression (depressive illness)
Rudzki 2017	Immune suppression of IgG response against dairy proteins in major depression	IgG response to dairy proteins, TNF-α, cortisol	depression
Rudolph 2017	Cognition-emotion interaction as a predictor of adolescent depressive symptoms	cognitive control (CC)	depressive symptoms
Roy 2017	Identification of MicroRNA-124-3p as a Putative Epigenetic Signature of Major Depressive Disorder	microRNAs (miRNAs)	major depressive disorder
Qiao 2017	Role of proBDNF and BDNF in dendritic spine plasticity and depressive-like behaviors induced by an animal model of depression	BDNF and proBDNF, chronic unpredicted mild stress	major depressive disorder
Platt 2017	A review of cognitive biases in youth depression: attention, interpretation and memory	cognitive biases, attention and interpretation biases	youth depression

Petkus 2017	Gene-environment interplay in depressive symptoms: moderation by age, sex, and physical illness	genetics, physical illness, environmental influences	depressive symptoms
Pereira 2017	Secondary to excessive melatonin synthesis, the consumption of tryptophan from outside the blood-brain barrier and melatonin over-signaling in the pars tuberalis may be central to the pathophysiology of winter depression	serotonin production and central hypothyroidism, melatonin production, circulating melatonin, tryptophan	winter depression
Pariante 2017	Why are depressed patients inflamed? A reflection on 20 years of research on depression, glucocorticoid resistance and inflammation	inflammation, HPA axis, early life trauma, maternal depression, infection, autoimmune disorder, genes	major depression
Normansell 2017	Negative interpretation bias as a mechanism of the relationship between rejection sensitivity and depressive symptoms	negative cognitive/ interpretation biases	depressive symptoms

Michalek 2017	Genetic predisposition to advanced biological ageing increases risk for childhood-onset recurrent major depressive disorder in a large UK sample	genetic predisposition to shorter telomere lengths	recurrent major depressive disorder
Macrynikola 2017	Positive future-oriented fantasies and depressive symptoms: Indirect relationship through brooding	fantasy engagement	depressive symptoms
Ma 2017	GNB3 and CREB1 gene polymorphisms combined with negative life events increase susceptibility to major depression in a Chinese Han population	genetic factors, (negative life events)	major depression
Liu 2017	The Role of Neural Plasticity in Depression: From Hippocampus to Prefrontal Cortex	neural plasticity, (stress, negative stimuli)	depression
Kvæl 2017	Associations between physical function and depression in nursing home residents with mild and moderate dementia: a cross-sectional study	muscle strength, balance, walking speed	depressive symptoms

Kuhlmann 2017	Serum brain-derived neurotrophic factor and depressive symptoms in coronary heart disease patients: Role of cognitive functions	BDNF	depressive symptoms
Krzak 2017	Does neurogenesis relate to depression and do antidepressants affect neurogenesis?	Brain mechanisms	depression
Kraus 2017	Serotonin and neuroplasticity - Links between molecular, functional and structural pathophysiology in depression	(Serotonin), neuroplasticity	Depression
Koopman 2017	Depressed gut? The microbiota-diet-inflammation trialogue in depression	gut microbiota, early life trauma, diet, inflammation	depression
Knyazev 2017	Suppression mediates the effect of 5-HTTLPR by stress interaction on depression	stress, (genetics, emotion regulation)	depression

Kim 2017	The Impacts of Social Support and Cognitive Function on Depression among Community-Dwelling Older Japanese Americans	social support, cognitive function, and socioeconomic status	depression
Jeon 2017	Inflammation-induced depression: Its pathophysiology and therapeutic implications	kynurenine pathway, (tryptophan, serotonin, inflammation)	depression
Haverkamp 2017	The role of tryptophan degradation in the association between inflammatory markers and depressive symptoms in chronic dialysis patients	inflammatory markers, (kynurenine pathway, KYN/TRP ratio)	depressive symptoms
Han 2017	Interactions between the vascular endothelial growth factor gene polymorphism and life events in susceptibility to major depressive disorder in a Chinese population	genetics, (negative life events)	major depressive disorder

Gulyaeva 2017	Interplay between Brain BDNF and Glutamatergic Systems: A Brief State of the Evidence and Association with the Pathogenesis of Depression	glutamatergic and BDNF systems	depressive disorders
Gardiner 2017	Large normal-range TBP and ATXN7 CAG repeat lengths are associated with increased lifetime risk of depression	genetic polymorphisms	lifetime depression
Fuhr 2017	Attachment, dysfunctional attitudes, self-esteem, and association to depressive symptoms in patients with mood disorders	attachment style	depressive symptoms
Everaert 2017	Individual differences in cognitive control over emotional material modulate cognitive biases linked to depressive symptoms	negative attention bias, cognitive control	depressive symptoms
Everaert 2017	Mapping the interplay among cognitive biases, emotion regulation, and depressive symptoms	Cognitive biases and emotion regulation	depressive symptoms

De Oliveira 2017	Social Determinants of Depression Among Hispanic Women	social determinants (education, health status, living with partner, [living with children, income, acculturation, health insurance status, employment status])	depression
Dantzer 2017	Role of the Kynurenine Metabolism Pathway in Inflammation-Induced Depression: Preclinical Approaches	kynurenine metabolism pathway, inflammation	depression
Connolly 2017	Rumination interacts with life stress to predict depressive symptoms: An ecological momentary assessment study	stress, rumination	depressive symptoms
Cissé 2017	Depressive-like behavior is elevated among offspring of parents exposed to dim light at night prior to mating	artificial nighttime lighting - light at night	Depressive-like behaviour
Chen 2017	Conscientiousness Moderates the Relationship Between Perceived Stress and Depressive Symptoms Among U.S. Chinese Older Adults	perceived stress	depressive symptoms

Caputi 2017	Do Feelings of Loneliness Mediate the Relationship between Sociocognitive Understanding and Depressive Symptoms During Late Childhood and Early Adolescence?	sociocognitive understanding	depression
Brown 2017	Social determinants of depression and suicidal behaviour in the Caribbean: a systematic review	social inequalities / determinants	depression
Zhu 2020	Depressive-like behaviors are induced by chronic liver injury in male and female mice	Chronic liver injury	Depressive-like behaviour
Zhong 2020	Epigenetic Modifications of GABAergic Interneurons Contribute to Deficits in Adult Hippocampus Neurogenesis and Depression-Like Behavior in Prenatally Stressed Mice	Prenatal stress	Depression phenotype

Zhang 2020	The Association between Dietary Patterns and Depressive Symptoms in Chinese Adults	eating patterns	depressive symptoms
Zajączkowska 2020	Polymorphism and expression of the Dvl3 gene in the etiology of depressive disorder	Dvl3 gene	depressive disorder
Yu 2020	Prenatal ethanol exposure enhances the susceptibility to depressive behavior of adult offspring rats fed a high-fat diet by affecting BDNF-associated pathway	prenatal ethanol exposure	depression
Wright 2020	New-onset depression among children, adolescents, and adults with hidradenitis suppurativa	hidradenitis suppurativa	depression
Winding 2020	Is bullying in adolescence associated with the development of depressive symptoms in adulthood?: A longitudinal cohort study	bullying	depressive symptoms
Wellman 2020	Secondhand Smoke Exposure and Depressive Symptoms in Children: A Longitudinal Study	secondhand smoke	depressive symptoms

Wang 2020	Meta-analysis of 5-hydroxytryptamine transporter gene promoter region polymorphism and post-stroke depression	5-hydroxytryptamine transporter gene promoter region (5-HTTLPR) gene polymorphism	post-stroke depression
Wang 2020	Gene–disease association study of tumor necrosis factor-α G-308A gene polymorphism with risk of major depressive disorder: A systematic review and meta-analysis	TNF-α G-308A gene polymorphism	poststroke, late-life, maternal, or major depression
Wang 2020	Integrating genome-wide association study and expression quantitative trait loci data identifies NEGR1 as a causal risk gene of major depression disorder	SNP	Major depressive disorder
Wang 2020	Advance in relationship between receptor gene abnormality and depression	genes	depression

Wang 2020	Variants in the upstream region of the insulin receptor substrate-1 gene is associated with major depressive disorder in the han chinese population	susceptible gene	major depressive disorder
Walker 2020	Acute exposure to low-level light at night is sufficient to induce neurological changes and depressive-like behavior	light at night (LAN)	depressive-like responses
Tonon 2020	How do stress, sleep quality, and chronotype associate with clinically significant depressive symptoms? A study of young male military recruits in compulsory service	stress, sleep, evening types	depressive symptoms
Tomita 2020	Spatial clustering of food insecurity and its association with depression: a geospatial analysis of nationally representative South African data, 2008-2015	food insecurity	depression

Sunley 2020	Ethnic and sex differences in the association of child maltreatment and depressed mood. The HELIUS study	emotional neglect, psychological abuse	depressed mood
Sparling 2020	Depression among women of reproductive age in rural Bangladesh is linked to food security, diets and nutrition	food security, diets and nutrition	depression
Sharma 2020	Association of placental tissue estrogen receptor alpha gene expression with postpartum depression in women at risk: A pilot study	ESRα gene	postpartum depression
Schnittker 2020	In-utero determinants of adult depression: evidence from the 1918 flu pandemic	gestational exposure to the 1918 flu pandemic	adult depression
Schiweck 2020	Childhood trauma, suicide risk and inflammatory phenotypes of depression: insights from monocyte gene expression	early stressors	depression
Sahasrabudhe 2020	Serum Vitamin D and Depressive Symptomatology among Boston-Area Puerto Ricans	serum 25(OH)D	depressive symptomatology

Roomaney 2020	Biopsychosocial predictors of symptoms of depression in a sample of South African women diagnosed with endometriosis	biopsychosocial predictors	depression symptoms
Rivet-Noor	The Role of Gut Mucins in the Etiology of Depression	stress, microbiome	depression
Rathbun 2020	Association between disease progression and depression onset in persons with radiographic knee osteoarthritis	Osteoarthritis (OA) disease progression	depression
Ramirez-Carrillo 2020	Disturbance in human gut microbiota networks by parasites and its implications in the incidence of depression	parasites/ microbiome	depression
Quinn 2020	Trying again (and again): Weight cycling and depressive symptoms in U.S. adults	weight cycling	depressive symptoms
Powell 2020	What explains the link between childhood ADHD and adolescent depression? Investigating the role of peer relationships and academic attainment	childhood ADHD	depression

Nishida 2020	Post-weaning folate deficiency induces a depression-like state via neuronal immaturity of the dentate gyrus in mice	folate deficiency	depressive symptoms
Mulugeta 2020	Relationship between Serum 25(OH)D and Depression: Causal Evidence from a Bi-Directional Mendelian Randomization Study	vitamin D	depression risk
Moustafa 2020	Depression Following Major Life Transitions in Women: A Review and Theory	life transitions	depression
Min 2020	The Impact of Spouse's Illness on Depressive Symptoms: The Roles of Spousal Caregiving and Marital Satisfaction	chronic illness	depression
Millender 2020	Associations between social determinants of health, perceived discrimination, and body mass index on symptoms of depression among young african american mothers	elevated BMI, discrimination	depression symptoms

Meyer 2020	Length Polymorphisms in the Angiotensin I-Converting Enzyme Gene and the Serotonin-Transporter-Linked Polymorphic Region Constitute a Risk Haplotype for Depression in Patients with Coronary Artery Disease	genetic variation	depressive symptoms
Medeiros 2020	Post-stroke depression: A 2020 updated review	female sex, history of psychiatric illness, large or multiple strokes, injuries in frontal/anterior areas or in the basal ganglia, stroke occurrence within the past year, poor social support, and pronounced disability	post-stroke depression
Manczak 2020	Water contaminant levels interact with parenting environment to predict development of depressive symptoms in adolescents	drinking water contaminants	depression
Madison 2020	Endotoxemia coupled with heightened inflammation predicts future depressive symptoms	endotoxemia, systematic inflammation	depressive symptoms

Liu 2020	The BDNF-FoxO1 Axis in the medial prefrontal cortex modulates depressive-like behaviors induced by chronic unpredictable stress in postpartum female mice	chronic unpredictable stress	depression-associated behaviours
Lin 2020	Is trade a blessing or a curse? A panel data analysis of the determinants of depressive disorders	international trade	depressive disorders
Li 2020	Association between dietary protein intake and the risk of depressive symptoms in adults	protein intake	depressive symptoms
Li 2020	Regulatory mechanisms of major depressive disorder risk variants	genetic variants	MDD risk
Li 2020	Linking childhood emotional abuse and adult depressive symptoms: The role of mentalizing incapacity	emotional abuse	depressive symptoms

Li 2020	Association between obstructive sleep apnea and risk of post-stroke depression: A hospital-based study in ischemic stroke patients	obstructive sleep apnea	post-stroke depression
Leonard 2020	Postpartum Perceived Stress Explains the Association between Perceived Social Support and Depressive Symptoms	social support, stress	depressive symptoms
Lee 2020	Comparison of prevalence and associated factors of depressive disorder between patients with head and neck cancer and those with lung cancer at a tertiary hospital in Taiwan: a cross-sectional study	self-harm history, educational level, stressor severity, anxiety severity	depression
Lansner 2020	Development of depression in patients with oral cavity cancer: a systematic review	treated for cancer	depressive symptoms
Kubacka 2020	Kynurenine pathway: The link between depressive disorders and inflammation	kynurenine pathway	depressive disorders

Koskinen 2020	From stress to depression: development of extracellular matrix-dependent cognitive impairment following social stress	stress	depressive-like state
Koga 2020	Elder Abuse and Depressive Symptoms: Which is Cause and Effect? Bidirectional Longitudinal Studies From the JAGES	abuse	depression
Kobezak 2020	Prospective associations between social anxiety and depression in youth: The moderating role of maternal major depressive disorder	social anxiety	depressive symptoms
Klimova 2020	The Impact of Nutrition and Intestinal Microbiome on Elderly Depression-A Systematic Review	nutrition	depressive symptoms

Kim 2020	The combined effect of socioeconomic status and metabolic syndrome on depression: the Korean National Health and Nutrition Examination Survey (KNHANES)	SES, metabolic syndrome	depression
Kautz 2020	Longitudinal changes of inflammatory biomarkers moderate the relationship between recent stressful life events and prospective symptoms of depression in a diverse sample of urban adolescents	Recent stressful life events	Depressive symptoms
Karl 2020	Precarious working conditions and psychosocial work stress act as a risk factor for symptoms of postpartum depression during maternity leave: results from a longitudinal cohort study	work-privacy conflict, low reward at work, and precarious working conditions	postpartum depression

Johar 2020	Evaluation of antenatal risk factors for postpartum depression: a secondary cohort analysis of the cluster-randomised GeliS trial	antenatal risk factors	postpartum depression
Hunt 2020	Effect of immune activation on the kynurenine pathway and depression symptoms - A systematic review and meta-analysis	immune-activating agents	depression
Hughes 2020	A possible mechanism underlying mood disorders associated with LUTS: Chronic bladder outlet obstruction causes NLRP3-dependent inflammation in the hippocampus and depressive behavior in rats	bladder outlet obstruction (BOO)	depressive behaviour
Ho 2020	Factors associated with depression in people with epilepsy: a retrospective case-control analysis	Female sex, refractoriness, and clonazepam use	depression

Hayashi 2020	Factors associated with symptoms of depression among pregnant women with gestational diabetes mellitus in Japan	diet	depression symptoms
Hashimoto 2020	Early life stress from allergic dermatitis causes depressive-like behaviors in adolescent male mice through neuroinflammatory priming	early life stress from allergic dermatitis	depressive behaviours
Haleem 2020	Glucocorticoids in the Physiological and Transcriptional Regulation of 5-HT1A Receptor and the Pathogenesis of Depression	5-HT1A receptors, glucocorticoids, among other factors	depression
Gutiérrez-Zotes 2020	Interaction between the functional SNP rs2070951 in NR3C2 gene and high levels of plasma corticotropin-releasing hormone associates to postpartum depression	genotypes	postpartum depression

Flouri 2020	Prenatal and childhood adverse life events, inflammation and depressive symptoms across adolescence	psychosocial stressors	depressive symptoms
Fang 2020	Cerebral Small-Vessel Disease and Risk of Incidence of Depression: A Meta-Analysis of Longitudinal Cohort Studies	Cerebral small-vessel disease features	depression
Dong 2020	Prevalence of Depressive Symptoms and Associated Factors among Internal Migrants with Tuberculosis: A Cross-Sectional Study in China	female gender, lower education, family dysfunction, poor doctor-patient communication, and TB-related stigma	depressive symptoms
Didone 2020	Factors associated with depressive symptoms in older adults in context of social vulnerability	Quality of life and social support, malnutrition, living alone, reporting pain and being female	depressive symptoms
Dadi 2020	Causal mechanisms of postnatal depression among women in Gondar town, Ethiopia: application of a stress-process model with generalized structural equation modeling	common mental disorders before pregnancy and low birth weight	Postnatal depression

Czaderny 2020	Risk factors for depression. New evidence on selenium deficiency and depressive disorders	dietary and non-dietary factors	depressive disorders
Chen 2020	Cataract and the increased risk of depression in general population: a 16-year nationwide population-based longitudinal study	cataract	depression
Chen 2020	Mitochondria could be a potential key mediator linking the intestinal microbiota to depression	gut microbiome	depression
Cavazos-Rehg 2020	Social and Economic Equity and Family Cohesion as Potential Protective Factors from Depression Among Adolescents Living with HIV in Uganda	social and economic equity	depression
Capuco 2020	Vitamin D and Depression in Women: A Mini-review	vitamin D	depression
Benjet 2020	Exposure to Armed Conflict in Childhood vs Older Ages and Subsequent Onset of Major Depressive Disorder	neighbourhood with beatings	major depressive disorder

Ayano 2020	Food insecurity and the risk of depression in people living with HIV/AIDS: a systematic review and meta-analysis	food insecurity	depression
An 2020	Parasympathetic predominance is a risk factor for future depression: A prospective cohort study	parasympathetic predominance	depression
Almazan 2020	Stroke, Epilepsy, and Antiepileptic Drugs as the Etiology of Major Depressive Disorder: A Case Report and Review	structural, electrophysiologic, and pharmacologic factors	post-stroke depression
Zhong 2019	Integration of GWAS and brain eQTL identifies FLOT1 as a risk gene for major depressive disorder	risk gene	major depressive disorder
Zhang 2019	Prenatal nicotine exposure induces depression-like behavior in adolescent female rats via modulating neurosteroid in the hippocampus	prenatal nicotine exposure	depression-like behaviour
Zeng 2019	Co-expression network analysis revealed that the ATP5G1 gene is associated with major depressive disorder	ATP5G1 gene	major depressive disorder

Ye 2019	Setting-Specific and Symptom-Specific Association between Secondhand Smoke Exposure and Depressive Symptoms	secondhand smoke	depressive symptoms
Xue 2019	Analysis of Influencing Factors of Poststroke Depression: Is Higher Body Mass Index Always a Risk Factor of Poststroke Depression?	National Institute of Health Stroke Scale score, the frequency of stroke, sleep duration, and BMI	poststroke depression
Wen 2019	The risk factors for depression in cancer patients undergoing chemotherapy: a systematic review	social support, anxiety, perceived stress, and self-efficacy	depression
Wassef 2019	Anaemia and depletion of iron stores as risk factors for postpartum depression: a literature review	Anaemia, iron-deficiency	postpartum depression
Wang 2019	Diversiform etiologies for poststroke depression	biological mechanism and social psychological mechanisms	poststroke depression
Wang 2019	The associations between screen time-based sedentary behavior and depression: a systematic review and meta-analysis	screen time-based sedentary behavior	depression

Wang 2019	Cross-sectional associations between long-term exposure to particulate matter and depression in China: The mediating effects of sunlight, physical activity, and neighborly reciprocity	air pollution	depression
Wang 2019	Association between negative life events and early adolescents' depression: The moderating effects of catechol-O-methyltransferase (COMT) gene val158met polymorphism and parenting behavior	negative life events	depression
Volz 2019	General self-efficacy as a driving factor of post-stroke depression: A longitudinal study	self-efficacy	post-stroke depression
Tiburcio Sainz	The association between substance use and depressive symptomatology in nursing university students in Mexico	substance use	depressive symptomatology

Tao 2019	Chronic Food Antigen-specific IgG-mediated Hypersensitivity Reaction as A Risk Factor for Adolescent Depressive Disorder	serum histamine	depressive disorders
Su 2019	Inflammation associated with chronic heart failure leads to enhanced susceptibility to depression	chronic heart failure	depression
Stroud 2019	The cortisol awakening response (CAR) interacts with acute interpersonal stress to prospectively predict depressive symptoms among early adolescent girls	cortisol awakening response, stress	depressive symptoms
Starr 2019	Serotonergic multilocus genetic variation moderates the association between major interpersonal stress and adolescent depressive symptoms: Replication and candidate environment specification	stress	depression

Saul 2019	Polymorphism in the serotonin transporter gene polymorphisms (5-HTTLPR) modifies the association between significant life events and depression in people with multiple sclerosis	Significant life events	multiple sclerosis depression
Santero 2019	Association between religiosity and depression varies with age and sex among adults in South America: Evidence from the CESCAS I study	Religiosity	depression
Ruiz 2019	Congruent relations between perceived neighbourhood social cohesion and depressive symptoms among older European adults: An East-West analysis	social cohesion	depressive symptoms
Rhee 2019	Longitudinal study of the relationship between lower urinary tract symptoms and depressive symptoms	lower urinary tract symptoms	depressive symptoms

Ren 2019	Associations of family relationships and negative life events with depressive symptoms among Chinese adolescents: A cross-sectional study	family relationships	depressive symptoms, life events
Raudsepp 2019	Brief report: Problematic social media use and sleep disturbances are longitudinally associated with depressive symptoms in adolescents	problematic social media use, sleep disturbances	depressive symptoms
Ranjit 2019	Predictive Association of Smoking with Depressive Symptoms: a Longitudinal Study of Adolescent Twins	cigarette smoking	depressive symptoms
Raffetti 2019	Longitudinal association between tobacco use and the onset of depressive symptoms among Swedish adolescents: the Kupol cohort study	cigarette smoking	depressive symptoms

Pramod 2019	Lifestyle and substance use-an important cause for treatment-resistant depression and major depressive disorders	lifestyle, substance use	depression
Pereira-Lima 2019	Residency Program Factors Associated With Depressive Symptoms in Internal Medicine Interns: A Prospective Cohort Study	Poor faculty feedback and inpatient learning experience, long work hours, and high institutional research rankings	depressive symptoms
Peppard 2019	Risk of depression in pregnant women with low-normal serum Vitamin B12	vitamin B12	depression
Ozgan 2019	Evaluation of the relationship between sexual functions and depressive symptoms among pregnant patients during the second trimester	sexual function	depressive symptoms
Miwata 2019	Performance Status Is a Risk Factor for Depression before the Diagnosis of Lung Cancer Patients	performance status	depression

Meng 2019	Dysmenorrhea increased the risk of postpartum depression in Chinese Han parturients	dysmenorrhea	postpartum depression
Mendes-Chiloff 2019	Depressive symptoms among the elderly in Sao Paulo city, Brazil: prevalence and associated factors (SABE Study)	health problems, dependence and family dysfunction	depressive symptoms
McQuaid 2019	Understanding the relation between early-life adversity and depression symptoms: The moderating role of sex and an interleukin-1β gene variant	early life adversity	depressive symptomatology
Malmir 2019	Metabolically healthy status and BMI in relation to depression: A systematic review of observational studies	health status, BMI	depression
Lu 2019	Inflammation and Risk of Depression in HIV: Prospective Findings From the Multicenter AIDS Cohort Study	immune activation	depression

Liu 2019	Mediating role of resilience in relationship between negative life events and depression among Chinese adolescents	negative life events	depression
Lin 2019	The association between functional dyspepsia and depression: a meta-analysis of observational studies	functional dyspepsia	depression
Libuda 2019	Vitamin D and the Risk of Depression: A Causal Relationship? Findings from a Mendelian Randomization Study	vitamin D	depressive symptoms, broad depression
Liang 2019	Exploring causal pathways linking cerebral small vessel diseases burden to poststroke depressive symptoms with structural equation model analysis	cerebral small vessel disease	Poststroke depressive symptoms
Li 2019	Adhering to a vegetarian diet may create a greater risk of depressive symptoms in the elderly male Chinese population	vegetarian diet	depressive symptoms

Lee 2019	Household food insecurity and breakfast skipping: Their association with depressive symptoms	low food security, breakfast skipping	depressive symptoms
Kong 2019	The association of endocannabinoid receptor genes (CNR1 and CNR2) polymorphisms with depression: A meta-analysis	CNR1rs1049353 or AAT triplet repeat polymorphism, CNR2rs2501432 polymorphism	depression
Kim 2019	Income Volatility and Depressive Symptoms among Elderly Koreans	income volatility	depressive symptoms
Kheirouri 2019	Dietary Inflammatory Potential and the Risk of Incident Depression in Adults: A Systematic Review	dietary inflammatory potential	depression
Khaled 2019	Prevalence and potential determinants of subthreshold and major depression in the general population of Qatar	migrant status, ethnicity	subthreshold and major depression
Hymas 2019	Predicting postpartum depression among adolescent mothers: A systematic review of risk	prior depression, familial social support	postpartum depression

Humphreys	DNA methylation of HPA-axis genes and the onset of major depressive disorder in adolescent girls: a prospective analysis	variation in the HPA axis at the level of the methylome	major depressive disorder
Hu 2019	Sugar-sweetened beverages consumption and the risk of depression: A meta-analysis of observational studies	sugar-sweetened beverages	depression
Hessel 2019	Acute exposure to violent neighborhood crime and depressive symptoms among older individuals in Colombia	violent crime exposure	depressive symptoms
Grases 2019	Possible relation between consumption of different food groups and depression	food groups	depression
Gao 2019	Association between earthquake experience and depression 37 years after the Tangshan earthquake: a cross-sectional study	earthquake experience	depression
Friberg 2019	Educational level and the risk of depression after prostate cancer	education level	depression

Easton 2019	Child Sexual Abuse and Depression in Late Life for Men: A Population-Based, Longitudinal Analysis	childhood sexual abuse	depressive symptoms
Duan 2019	Association between serum retinoic acid levels and risk of post-stroke depression in patients with ischemic stroke	retinoic acid	poststroke depression
Ding 2019	Correlation between C3435T Locus of ABCB1 Gene and Poststroke Depression in China	C3435T gene polymorphism	poststroke depression
de Paula 2019	Preterm birth as a risk factor for postpartum depression: A systematic review and meta-analysis	preterm birth	postpartum depression
de Lima 2019	Factors related to depressive symptoms in older adult patients with type 2 Diabetes Mellitus	being female, being single, being illiterate, having poor overall health, using a walking aid, having higher body mass index, having a greater intensity of dizziness, exhibiting functional impairment, and having gait and cognition deficits.	depressive symptoms

Carpena 2019	Sociodemographic, behavioral, and health-related risk factors for depression among men and women in a southern Brazilian city	socioeconomic disadvantages, physical inactivity, stress	major depressive episodes
Byrne 2019	Sleep Disorders and Risk of Incident Depression: A Population Case-Control Study	sleep disorder	depression
Brown 2019	Declining Skeletal Muscle Mitochondrial Function Associated With Increased Risk of Depression in Later Life	skeletal muscle mitochondrial function	depressive symptoms
Bonful 2019	Sociodemographic correlates of depressive symptoms: a cross-sectional analytic study among healthy urban Ghanaian women	age, education, employment status	depression
Bolzetta 2019	The Relationship between Dietary Vitamin K and Depressive Symptoms in Late Adulthood: A Cross-Sectional Analysis from a Large Cohort Study	vitamin K	depressive symptoms

Boldarine 2019	High-fat diet intake induces depressive-like behavior in ovariectomized rats	high-fat intake	depression
Boing 2019	Factors associated with depression symptoms in women after breast cancer	Age, educational attainment, diagnosis of other diseases, type of surgery, lymphedema, self-esteem, and body image	depression symptoms
Azizi 2019	Depressive symptoms and associated factors among Iranian women at midlife: a community-based, cross-sectional study	Marital relationship dissatisfaction, vasomotor symptoms, housing insecurity, obesity, age	depressive symptoms
Avinun 2019	A polygenic score for body mass index is associated with depressive symptoms via early life stress: Evidence for gene-environment correlation	BMI polygenic scores	depressive symptoms
Arafa 2019	Gestational diabetes and risk of postpartum depressive symptoms: A meta-analysis of cohort studies	gestational diabetes	Postpartum depressive symptoms
Albrecht 2019	Risk of Depression after Traumatic Brain Injury in a Large National Sample	History of neuropsychiatric disturbances, age, gender	depression

Zhang 2018	Cross-sectional associations of objectively assessed neighbourhood attributes with depressive symptoms in older adults of an ultra-dense urban environment: the Hong Kong ALECS study	destinations and social networks, public transport density and associated traffic volumes	depressive symptoms
Zetterstrom 2018	Online sexual victimization in youth: predictors and cross-sectional associations with depressive symptoms	Online unwanted sexual solicitation victimization	depressive symptoms
Yamamoto 2018	Perceived Neighborhood Conditions and Depression: Positive Local News as a Buffering Factor	neighbourhood conditions	depression
Xuan 2018	Type 2 diabetes is causally associated with depression: a Mendelian randomization analysis	type 2 diabetes	depression
Xu 2018	Exploration of the association between dietary fiber intake and depressive symptoms in adults	dietary fiber intake	depressive symptoms

Wu 2018	Child maltreatment and adult depressive symptoms: Roles of self-compassion and gratitude	Psychological maltreatment, neglect, sexual abuse, physical abuse	depressive symptoms
Wilson 2018	Hysterectomy and incidence of depressive symptoms in midlife women: the Australian Longitudinal Study on Women's Health	hysterectomy	depressive symptoms
Williams 2018	The relationship between caregiver burden and depressive symptoms in Ontario home care clients	caregiver burden	depressive symptoms
Werneck 2018	Associations between TV viewing and depressive symptoms among 60,202 Brazilian adults: The Brazilian national health survey	TV viewing	depression
Wang 2018	The Relationship between Air Pollution and Depression in China: Is Neighbourhood Social Capital Protective?	air pollution	depressive symptoms

Virtanen 2018	Long working hours and depressive symptoms: systematic review and meta-analysis of published studies and unpublished individual participant data	working hours	depressive symptoms
Vancampfort 2018	Sedentary behavior and depressive symptoms among 67,077 adolescents aged 12-15 years from 30 low- and middle-income countries	sedentary behaviour	depressive symptoms
Sainsbury 2018	The relationship between gluten free diet adherence and depressive symptoms in adults with coeliac disease: A systematic review with meta-analysis	gluten free diet	depressive symptoms
Rui 2018	Meta-analysis on the Association Between Norepinephrine Transporter Gene rs2242446, rs5569 Polymorphisms and Risk of Major Depressive Disorder	NET gene rs5569 polymorphism	major depressive disorder

Ren 2018	Is social support a cause or consequence of depression? A longitudinal study of adolescents	social support	depression
Poole 2018	Major depressive disorder prevalence and risk factors among Syrian asylum seekers in Greece	gender, children, time in asylum	major depressive disorder
Pittampalli 2018	Does Vitamin D deficiency cause depression?	vitamin D	depression
Park 2018	Association of depression with socioeconomic status, anticardiolipin antibodies, and organ damage in patients with systemic lupus erythematosus: results from the KORNET registry	current smoking status, CL-status, and SLICC damage index score, education, income	depression
Nathanson 2018	Antenatal diet and postpartum depressive symptoms: A prospective study	antenatal diet	depressive symptoms postpartum
Mossakowska-Wójcik 2018	The importance of TCF4 gene in the etiology of recurrent depressive disorders	TCF4 gene	recurrent depressive disorder

Miki 2018	Longitudinal adherence to a dietary pattern and risk of depressive symptoms: the Furukawa Nutrition and Health Study	diet pattern	depressive symptoms
Mahenge 2018	Adverse childhood experiences and intimate partner violence during pregnancy and their association to postpartum depression	Adverse childhood experiences, intimate partner violence	postpartum depression
Luo 2018	Education as a moderator in the effect of diabetes on depressive symptoms in Chinese middle-aged and older adults: A population-based longitudinal study	diabetes	depressive symptoms
Liu 2018	Antepartum urinary tract infection and postpartum depression in Taiwan - a nationwide population-based study	urinary tract infection	postpartum depression
Liu 2018	The metabolic factor kynurenic acid of kynurenine pathway predicts major depressive disorder	kynurenine pathway factors	major depressive disorder

Leonard 2018	Inflammation and depression: a causal or coincidental link to the pathophysiology?	inflammation	depression
Lam 2018	Genotype-dependent associations between serotonin transporter gene (SLC6A4) DNA methylation and late-life depression	SLC6A4 methylation	late-life depression
Lahav-Kadmiel 2018	Bullying victimization and depressive symptoms in adolescence: The moderating role of parent-child conflicts among boys and girls	bullying victimization	depressive symptoms
Kalmbach 2018	Nocturnal insomnia symptoms and stress-induced cognitive intrusions in risk for depression: A 2-year prospective study	insomnia	depression
Jorgensen 2018	Higher dietary inflammation is associated with increased odds of depression independent of Framingham Risk Score in the National Health and Nutrition Examination Survey	proinflammatory diet	depressive symptoms

Iliadis 2018	Women with prolonged nausea in pregnancy have increased risk for depressive symptoms postpartum	prolonged nausea	depressive symptoms
Huang 2018	Dose-Response Relationships between Second-Hand Smoke Exposure and Depressive Symptoms among Adolescents in Guangzhou, China	second-hand smoke	depressive symptoms
Hosseini 2018	The Prevalence of Anemia and its Association with Depressive Symptoms among Older Adults in North of Iran	anemia	depressive symptoms
Hamano 2018	Is familial risk for depression confounded by individual and familial socioeconomic factors and neighborhood environmental factors? A 7-year follow-up study in Sweden	family history of depression	depression
Hajek 2018	Impact of falls on depressive symptoms among the oldest old: Results from the AgeQualiDe study	falls	depressive symptoms

Guintivano 2018	Predictors of Postpartum Depression: A Comprehensive Review of the Last Decade of Evidence	psychiatric history, adverse life events	depression
Greenwood 2018	Insulin resistance is associated with depression risk in polycystic ovary syndrome	insulin resistance	depression
Graham	The association between diabetes and depressive symptoms varies by quality of diabetes care across Europe	diabetes	depressive symptoms
Gershon 2018	Victimization and traumatic stress: Pathways to depressive symptoms among low-income, African-American girls	victimization and traumatic stressors	depressive symptoms
Gayman 2018	Risk and Protective Factors for Depressive Symptoms Among African American Men: An Application of the Stress Process Model	stress process variables, neighbourhood income	depressive symptoms
Garcia-Esquinas 2018	Moderate alcohol drinking is not associated with risk of depression in older adults	alcohol consumption	depression

Flores 2018	The Association Between Past Sexual Abuse and Depression in Older Adults From Colombia	sexual abuse, history of being displaced by violence, socioeconomic status, education, self-rated health status, self-rated memory	depression
Fan 2018	Risk of depressive disorder among patients with head and neck cancer: A nationwide population-based study	head and neck cancer	depressive disorder
Dayan 2018	The Relationship between Gestational Weight Gain and Postpartum Depression in Normal and Overweight Pregnant Women	gestational weight gain	postpartum depression
Czarny 2018	Single-nucleotide polymorphisms of uracil-processing genes affect the occurrence and the onset of recurrent depressive disorder	SNPs localized in the uracil-processing genes	depression
Clevesy 2018	Is There a Relationship Between Gestational Diabetes and Perinatal Depression?	gestational diabetes mellitus	perinatal depression

Cheng 2018	Combination of High-Sensitivity C-Reactive Protein and Homocysteine Predicts the Post-Stroke Depression in Patients with Ischemic Stroke	high-sensitivity C-reactive protein, homocysteine	poststroke depression
Chen 2018	The association between glaucoma and risk of depression: a nationwide population-based cohort study	glaucoma	depression
Chen 2018	The association between neutrophil-to-lymphocyte ratio and post-stroke depression	neutrophil-to-lymphocyte ratio	poststroke depression
Cattaneo 2018	FoxO1, A2M, and TGF-β1: three novel genes predicting depression in gene X environment interactions are identified using cross-species and cross-tissues transcriptomic and miRNomic analyses	early life emotional stress	depression
Bertoni 2018	Depressive symptoms and muscle weakness: A two-way relation?	dynapenia	depressive symptoms

Ayalon 2018	Perceived Age Discrimination: A Precipitator or a Consequence of Depressive Symptoms?	age discrimination	depressive symptoms
Yang 2017	Lucky gene 5-HTTLPR and postpartum depression: A systematic review	5-HTTLPR polymorphism	postpartum depression
Xu 2017	Cesarean section and risk of postpartum depression: A meta-analysis	cesarian section	postpartum depression
Xu 2017	The interaction of miR-34b/c polymorphisms and negative life events increases susceptibility to major depressive disorder in Han Chinese population	miR-34b/c, negative life events	major depressive disorder
Wirth 2017	The Dietary Inflammatory Index, shift work, and depression: Results from NHANES	dietary inflammation	depressive symptoms
Varela 2017	Association between gestational diabetes and perinatal depressive symptoms: evidence from a Greek cohort study	gestational diabetes mellitus	depressive symptoms

Uddin 2017	Adolescent depression linked to socioeconomic status? Molecular approaches for revealing premorbid risk factors	SES, changes in 5mC, changes in brain function	depression
Shin 2017	Association of TNF-alpha G-308A gene polymorphism with depression: A meta-analysis	TNF-α G-308A polymorphism	depression
Sarubin 2017	The sex-dependent role of the glucocorticoid receptor in depression: variations in the NR3C1 gene are associated with major depressive disorder in women but not in men	GR-related genes	depression
Recto 2017	Psychosocial Risk Factors for Perinatal Depression among Female Adolescents: A Systematic Review	psychosocial risk factors	perinatal depression
Ortiz 2017	Determinants associated with chronic and incident depression in Mexican older adults	poverty, loneliness	depression

Ojagbemi 2017	Depression after Stroke in Sub-Saharan Africa: A Systematic Review and Meta-Analysis	education, cognitive impairment, physical disability, quality of life, marital status	poststroke depression
Neumann 2017	Recollections of Emotional Abuse and Neglect in Childhood as Risk Factors for Depressive Disorders and the Need for Psychotherapy in Adult Life	emotional abuse, neglect, attachment anxiety	depression
Nelson 2017	Childhood maltreatment and characteristics of adult depression: meta-analysis	Childhood maltreatment, especially emotional abuse and neglect	treatment-resistant depression
Nam 2017	The synergistic effect of breastfeeding discontinuation and cesarean section delivery on postpartum depression: A nationwide population-based cohort study in Korea	Breastfeeding discontinuation and cesarean section delivery	postpartum depression
Najman 2017	Maternal depression and family adversity: Linked pathways to offspring depression?	adverse life events, maternal depression	depression

Modalsli 2017	The association between the clinical diversity of psoriasis and depressive symptoms: the HUNT Study, Norway	psoriasis	depression
Meng 2017	Predictors of early-onset post-ischemic stroke depression: a cross-sectional study	NIHSS, HAMD scores, dopamine level, 5-hydroxytryptamine level, tumor necrosis factor-alpha level, nerve growth factor level	post-ischemic stroke depression
Markkula 2017	Predictors of new-onset depressive disorders - Results from the longitudinal Finnish Health 2011 Study	depressive symptoms, anxiety disorders, trust, childhood adversities	depressive disorders
Madsen 2017	Job strain as a risk factor for clinical depression: systematic review and meta-analysis with additional individual participant data	job strain	clinical depression
Ma 2017	GNB3 and CREB1 gene polymorphisms combined with negative life events increase susceptibility to major depression in a Chinese Han population	alleles	major depression

Liu 2017	Social defeat stress causes depression-like behavior with metabolite changes in the prefrontal cortex of rats	social defeat	depression-like behaviour
Liu 2017	Risk factors for postpartum depression among Chinese women: path model analysis	mobile phone use, maternal age, gestational hypertensive disorder, mode of feeding, infant weight	postpartum depression
Liu 2017	The YWHAE gene confers risk to major depressive disorder in the male group of Chinese Han population	risk gene	major depressive disorder
Li 2017	Dietary zinc and iron intake and risk of depression: A meta-analysis	dietary zinc and iron	depression
Li 2017	Dietary patterns and depression risk: A meta-analysis	dietary pattern	depression
Li 2017	Maternal history of child maltreatment and maternal depression risk in the perinatal period: A longitudinal study	maternal history of child physical and emotional neglect	perinatal depression
Leung 2017	Predictors of Postpartum Depression in Partnered Mothers and Fathers from a Longitudinal Cohort	household income, prenatal depressive symptoms, social support stressful life events, smoking	postpartum depression

Lee 2017	Gender differences in the trajectories and the risk factors of depressive symptoms in later life	physical health status, employment, social participation	depressive symptoms
Lee 2017	Depression and Its Associated Factors Among Rural Diabetic Residents	gender, level of education, family support, physical activity, diabetic diet, activity levels, sleep	depression
Lac 2017	Association between religious practice and risk of depression in older people in the subacute setting	intrinsic religiosity, cognition	depression scores
Koukounari 2017	Pathways from maternal depression to young adult offspring depression: an exploratory longitudinal mediation analysis	maternal depression	depression
Kang 2017	Relationship between interleukin-1beta and depressive disorder after acute coronary syndrome	interleukin-1beta	depressive disorder
Joshi 2017	Pathways from neighborhood poverty to depression among older adults	neighbourhood poverty (and homicide)	depression

James 2017	Built Environment and Depression in Low-Income African Americans and Whites	built environment	depression
Ihongbe 2017	Do Successive Preterm Births Increase the Risk of Postpartum Depressive Symptoms?	preterm birth	Postpartum depressive symptoms
Han 2017	Interactions between the vascular endothelial growth factor gene polymorphism and life events in susceptibility to major depressive disorder in a Chinese population	interactions between the VEGF rs2010963 polymorphism and environment	major depressive disorder
Ha 2017	Can Obesity Cause Depression? A Pseudo-panel Analysis	BMI	depression
Groger 2017	Causes of Depression in Sarcoidosis	inflammation meditators, fatigue, burdensome and chronic course of sarcoidosis, steroids	depression
Gorlova 2017	Dynamics of the Development of Depressive-Like State in Rats Stressed by Chronic Exposure of Ultrasound of Variable Frequency	ultrasound	depressive-like state

Gardiner 2017	Huntingtin gene repeat size variations affect risk of lifetime depression	polymorphism	depression
Dean 2017	The neurobiology of depression: An integrated view	neurotransmission, HPA axis abnormalities	depression
Cross-Denny 2017	Using the Social Determinants of Health as a Framework to Examine and Address Predictors of Depression in Later Life	health, social support	depression in later life
Cabello 2017	The role of unhealthy lifestyles in the incidence and persistence of depression: a longitudinal general population study in four emerging countries	smoking, physical activity, alcohol drinking	depression
Bhandari 2017	Association of Inflammatory Bowel Disease (IBD) with Depressive Symptoms in the United States Population and Independent Predictors of Depressive Symptoms in an IBD Population: A NHANES Study	IBD, age, marital status	depressive symptoms

Beshai 2017	Minding the gap: Subjective relative deprivation and depressive symptoms	subjective relative deprivation	depression symptoms
Babkair 2017	Risk Factors for Poststroke Depression: An Integrative Review	stroke severity, cognitive impairment, physical disability, functional dependency	poststroke depression
Angerer 2017	Night Work and the Risk of Depression	nighttime shift work	depression
Andreeva 2017	Dyslipidemia as a Potential Moderator of the Association between Hearing Loss and Depressive Symptoms	hearing/hearing loss	depressive symptoms
Amutah-Onukagha 2017	An Examination of the Sociodemographic and Health Determinants of Major Depressive Disorder Among Black Women	poverty, smoking status	major depressive disorder
Almquist 2017	Associations between social support and depressive symptoms: social causation or social selection-or both?	social support	depressive symptoms
Alhasanat 2017	Risk For Postpartum Depression Among Immigrant Arabic Women in the United States: A Feasibility Study	social support, maternity blues	postpartum depressive symptoms

Adjibade 2017	The Inflammatory Potential of the Diet Is Associated with Depressive Symptoms in Different Subgroups of the General Population	inflammatory potential of diet, smoking	depressive symptoms
Tang 2020	Impaired ATP Release from Brain Astrocytes May be a Cause of Major Depression	stress, ATP	major depressive disorder
Cernadas 2020	Postpartum depression: Risks and early detection	mood factors	postpartum depression
Lee 2018	Assessing the relative contribution of social exclusion, income-poverty, and financial strain on depressive symptoms among older people in Hong Kong	social exclusion, financial strain	depressive symptoms
Weissman 2018	Risks for Major Depression: Searching for Stable Traits	cortical thinning	depressive illness
Vallerand 2018	Risk of depression among patients with acne in the U.K.: a population-based cohort study	acne	depression

Reding 2017	Perimenopausal depression and early menopause: cause or consequence?	premenopausal depression, past antidepressant use, stressful life events, vasomotor symptoms, weight, smoking status, ethnicity, education, estadiol secretion, reproductive lifespan and estrogen	perimenopausal depression
