# Supplementary figures and images for: Biological, Psychological, and Social Determinants of Depression: A Review of Recent Literature
Source: Brain Sci. 2021 Dec 10;11(12):1633. doi: 10.3390/brainsci11121633 (PMC8699555; doi:10.3390/brainsci11121633)

## Conceptual Framework: Determinants of Depression

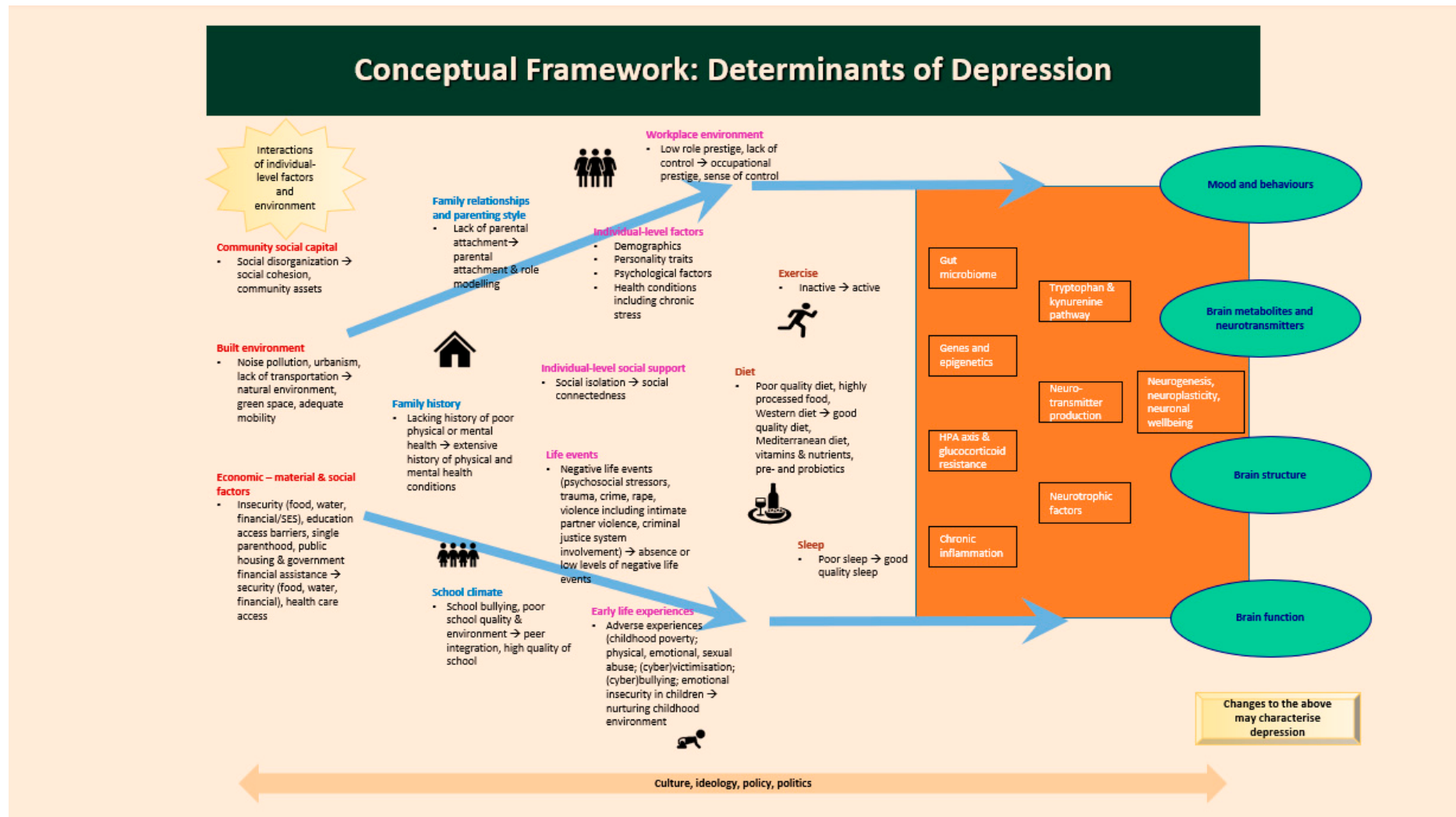

Figure S1. Determinants of depression

Supplement: Supplementary file 1 [file brainsci-11-01633-s001.zip › Supplementary Figure S1.pdf]
